# Modelling lung infection with *Klebsiella pneumoniae* after murine traumatic brain injury

**DOI:** 10.1186/s12974-024-03093-9

**Published:** 2024-05-08

**Authors:** Ali Shad, Sarah S. J. Rewell, Matthew Macowan, Natasha Gandasasmita, Jiping Wang, Ke Chen, Ben Marsland, Terence J. O’Brien, Jian Li, Bridgette D. Semple

**Affiliations:** 1 Department of Neuroscience, The School of Translational Medicine, Monash University, Level 6 Alfred Centre, 99 Commercial Rd, Melbourne, VIC 3004 VIC Australia; 2 Alfred Health, Prahran, VIC Australia; 3 Department of Immunology, The School of Translational Medicine, Monash University, Melbourne, VIC Australia; 4 GIN Discovery Program, The School of Translational Medicine, Monash University, Melbourne, VIC Australia; 5 Department of Microbiology, Monash Biomedical Discovery Institute, Monash University, Clayton, VIC Australia; 6 Department of Medicine (Royal Melbourne Hospital), University of Melbourne, Parkville, VIC Australia

**Keywords:** Bacteria, Pneumonia, Hospital-acquired infection, Inflammation, Controlled cortical impact

## Abstract

Pneumonia is a common comorbidity in patients with severe traumatic brain injury (TBI), and is associated with increased morbidity and mortality. In this study, we established a model of intratracheal *Klebsiella pneumoniae* administration in young adult male and female mice, at 4 days following an experimental TBI, to investigate how *K. pneumoniae* infection influences acute post-TBI outcomes. A dose-response curve determined the optimal dose of *K. pneumoniae* for inoculation (1 x 10^6 colony forming units), and administration at 4 days post-TBI resulted in transient body weight loss and sickness behaviors (hypoactivity and acute dyspnea). *K. pneumoniae* infection led to an increase in pro-inflammatory cytokines in serum and bronchoalveolar lavage fluid at 24 h post-infection, in both TBI and sham (uninjured) mice. By 7 days, when myeloperoxidase + neutrophil numbers had returned to baseline in all groups, lung histopathology was observed with an increase in airspace size in TBI + *K. pneumoniae* mice compared to TBI + vehicle mice. In the brain, increased neuroinflammatory gene expression was observed acutely in response to TBI, with an exacerbated increase in *Ccl2* and *Hmox1* in TBI + *K. pneumoniae* mice compared to either TBI or *K. pneumoniae* alone. However, the presence of neuroinflammatory immune cells in the injured brain, and the extent of damage to cortical and hippocampal brain tissue, was comparable between *K. pneumoniae* and vehicle-treated mice by 7 days. Examination of the fecal microbiome across a time course did not reveal any pronounced effects of either injury or *K. pneumoniae* on bacterial diversity or abundance. Together, these findings demonstrate that *K. pneumoniae* lung infection after TBI induces an acute and transient inflammatory response, primarily localized to the lungs with some systemic effects. However, this infection had minimal impact on secondary injury processes in the brain following TBI. Future studies are needed to evaluate the potential longer-term consequences of this dual-hit insult.

**Supplementary Information:**

The online version contains supplementary material available at 10.1186/s12974-024-03093-9.

## Background

Traumatic brain injury (TBI) continues to represent a major global public health burden [1]. Acutely post-injury, the hospitalization of individuals with severe TBI entails substantial in-hospital healthcare utilization, with higher costs associated with greater TBI severity, increased length of stay, and surgical interventions [2, 3]. Hospital-acquired infections pose a secondary complication for severe TBI patients, which drive higher healthcare costs by increasing the length of stay in intensive care, hospital, and in-patient rehabilitation, as well as increasing the frequency of hospital re-admission and likelihood of unfavorable long-term outcomes [4–7].

Hospital-acquired or ventilator-acquired pneumonia is the most commonly-reported infection in this population, affecting approximately 40% of severe TBI patients [8–14]. Susceptibility to pneumonia is heightened after severe TBI due to the frequent need for tracheal intubation and ventilator support, alongside injury-induced immunosuppression [15]. However, prophylactic use of antibiotics is not typically recommended as most studies have failed to demonstrate efficacy at improving outcomes [14, 16].

The most common pathogens that cause nosocomial infections in TBI patients are opportunistic Gram-negative bacteria such as *Klebsiella pneumoniae*, and Gram-positive *Staphylococcus aureus* [17–20]. *K. pneumoniae* is commonly detected in ventilator-associated pneumonia, where it causes severe acute lung damage and inflammation leading to respiratory failure in immunocompromised patients, including those with a TBI or spinal cord injury [21–25]. *K. pneumoniae* is highly virulent and increasingly a concern due to multidrug resistance [26], with carbapenem-resistant *K. pneumoniae* in particular being identified by the World Health Organization as a ‘Critical’ priority for the development of new antibiotics [27].

However, despite clinical evidence that bacterial infections lead to worse outcomes for TBI patients, we currently have an incomplete understanding of the biological mechanisms that contribute to an additional burden for the affected patient. An increasing appreciation of TBI-induced immune deficiencies [15, 28], and bi-directional communication between the acutely-injured brain and lungs [29, 30], provide new directions for targeted therapeutics to improve patient outcomes. Limited preclinical mouse models have examined the consequences of an infection following TBI [as recently reviewed: [31]. For example, intratracheal infection with *Pseudomonas aeruginosa* led to increased mortality and reduced lung bacterial clearance in mice that had sustained a severe TBI 24 h prior (via the controlled cortical impact (CCI) model), compared to similarly-infected uninjured mice [32]. In another study, Doran and colleagues administered intranasal *Streptococcus pneumoniae* at 3- or 60-days after moderate CCI, and reported higher mortality, poorer functional outcomes, and increased lung and neuroinflammation in mice with the combined insult [33]. By contrast, Vermeij and colleagues found that the combined insult of an intratracheal infection with *S. aureus* and a mild weight-drop injury in male rats did not lead to worse outcomes [34].

The consequences of lung infection with *K. pneumoniae* after TBI have not previously been examined in a preclinical model. Others have shown that intratracheal inoculation of naive mice with live *K. pneumoniae* leads to the rapid expansion of bacteria in the lungs within the first 24 h, leading to lung pathology, reduced pulmonary function, and associated morbidity [35–37], similar to the effects of *K. pneumoniae* infection in humans. In the current study, we sought to establish a new model of *K. pneumoniae*-mediated lung infection after TBI, to probe the effects of a secondary immune challenge on TBI outcomes over a sub-acute period (the first week) post-infection. We hypothesized that *K. pneumoniae* infection would result in poorer outcomes in TBI mice via increased immune activation and inflammation. We also examined the gut microbiome in this context, in light of recent evidence of complex bi-directional interactions between the brain, lungs and gut after brain injury [38–40].

## Methods

### Animals and ethics

All animal experiments were conducted following approval from the local Alfred Research Alliance Animal Ethics Committee (#P8032) as well as the Animal Care and Use Review Office (ACURO) from the Office of Research Protections, US Department of Defense, and carried out in accordance with these approved standards as well as the Australian Code for the Care and Use of Laboratory Animals as stipulated by the National Health and Medical Research Council of Australia (NHMRC). Male and female C57Bl/6J mice were sourced from the Walter and Eliza Hall Institute of Medical Research in Melbourne, Australia, and habituated for one week prior to the commencement of experiments. Animal experiments were performed within QC2 facilities at the Monash Research Animal Precinct in Clayton, Australia. Mice were group housed as same-sex littermates (2–6/cage; mix of experimental conditions per cage) in Optimice® individually-ventilated cages under a 12-h light/dark cycle with continuous access to food and water.

### Controlled cortical impact (CCI) model of TBI

Moderate-severe experimental TBI was induced in 10–12 week old mice using the CCI model, as previously described [41]. Briefly, anesthesia was induced using 4% isoflurane in oxygen, and maintained via a nose cone at 1.5%. All animals received buprenorphine (0.05 mg/kg in saline; s.c. flank) and bupivacaine (1 mg/kg in saline; s.c. scalp) for pain relief before surgery commenced, and 0.5 mL 0.9% saline at the end of the procedure to provide additional hydration. Animals were stabilized in a stereotaxic frame, and a 3.5 mm diameter craniotomy was made over the exposed left parietal bone. An electronic controlled cortical impactor device (eCCE-6.3; Custom Design and Fabrication Inc., Sandston, VA) delivered an injury using a 3 mm rounded impactor tip, at 4.5 m/s, 1.7 mm depth and 150 ms duration. Sham animals underwent an identical surgical procedure, without delivery of the impact. Following CCI (or sham surgery), the skin incision was sutured and antiseptic solution applied. Animals were allowed to recover in individual cages on a heat mat before being returned to their home cage.

### *K. pneumoniae* administration

*K. pneumoniae* ATCC 15,380 freeze-dried cultures were sourced from In Vitro Technologies (Noble Park, VIC, Australia), then cultivated onto nutrient agar plates (Medium Preparation Unit, University of Melbourne, Victoria, Australia) at 37 °C overnight. For the initial culture, a maximum number of *K. pneumoniae* colonies were collected and immersed in cation-adjusted Mueller-Hinton broth (CAMHB) for an additional 24 h incubation at 37 °C, while shaking at 180 rpm [42]. Mid-logarithmic-phase cultures were then prepared with a 3 h incubation in fresh CAMHB. The bacterial concentration (colony forming units (CFU)/mL) was determined by measuring the optical density (OD) at 600 nm and diluted to a working solution.

**Fig. 1:**
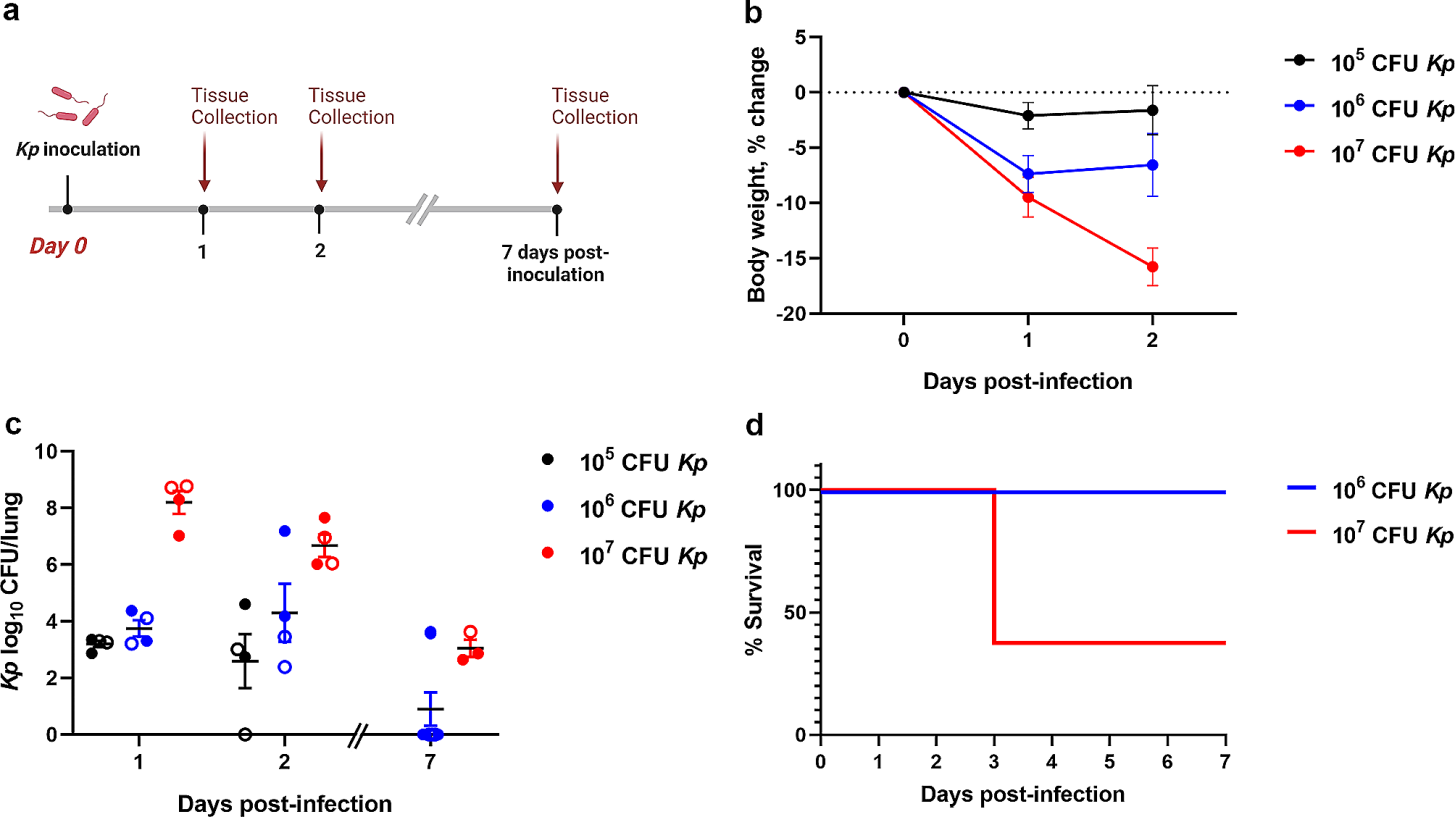
Dose-dependent responses to *K. pneumoniae* in Naïve mice. *K. pneumoniae* (*Kp*) or vehicle solution was administered intratracheally to naïve young adult mice (**a**). Changes in body weight (**b**), lung bacterial load (**c**) and % survival (**d**) were evaluated, with a dose of 10^6^ CFU found to be optimal. Mean ± SEM; CFU = colony-forming unit. *n* = 8/group (4 male and 4 female) for % survival (b). In (c), males are indicated by closed circles and females by open circles

An initial series of pilot studies were conducted to determine the optimal dose of *K. pneumoniae* via intratracheal inoculation to achieve an appropriate lung infection model, using naïve male and female mice. Inoculation was performed on day 0 followed by tissue collection at 24 h, 48 h and 7 d to evaluate changes in body weight, sickness symptoms, survival, and lung bacterial load (Fig. 1a), and testing 4 doses of K. pneumoniae: 1 × 10^4^, 1 × 10^5^, 1 × 10^6^ and 1 × 10^7^ CFU. A separate pilot cohort (*n* = 4/group) was used to compare sterile saline vs. broth (the vehicle for *K. pneumoniae* inoculation).

For the remaining experiments, mice were randomized for inoculation with 10^6^ CFU *K. pneumoniae* or vehicle on day 4 post-injury, as hospital-acquired infections are most common during the first week after a TBI [21, 43]. Following anesthesia with 2–4% inhaled isoflurane, an experienced technician used a MicroSprayer® Aerosolizer (Penn-Century, Philadelphia, PA, USA) to administer 25 µL bacterial suspension or vehicle solution directly into the trachea [44]. Following recovery from the procedure, mice were closely monitored post-treatment for sickness behavior, general appearance, and weight loss over the time course.

Additionally, five-fold serial dilutions of both *K. pneumoniae* and vehicle samples were spiral plated and incubated overnight at 37 °C on nutrient agar plates to verify the CFU/mL in the administered inoculum, using ProtoCOL 3 software (Synbiosis, USA).

### Open field locomotor activity

At 4 h, 24 h, and 7 d after inoculation, all mice underwent an open field test to evaluate locomotor activity and anxiety-like behavior [41]. Mice were individually placed in an empty arena (400 mm W x 400 mm D x 300 mm H) and allowed free exploration for 10 min. Activity was recorded using an overhead camera and analyzed using TopScan Lite v2.0.0 software (CleverSys Inc., Reston, VA, USA), to quantify the total distance travelled and the proportion of time spent in a predefined center zone (70% of the total arena size).

### Tissue collection for histological analyses

At 7 days post-infection, mice were humanely euthanized with 160 mg/kg i.p. sodium pentobarbitone (Lethabarb®, Virbac, Australia). Cardiac puncture was performed to collect blood samples for serum cytokine analysis, and the spleen was removed and weighed. A subset of mice were transcardially perfused with 0.9% saline then 4% paraformaldehyde (PFA) for fixation. Lungs were inflated in situ with 4% PFA then removed and post-fixed, and brains were collected whole. Samples were transferred to 70% ethanol and sent to the Monash Histology Platform (Monash University, Clayton, Australia) for paraffin processing and embedding. Coronal brain sections of 7 μm-thickness spanning approximately − 0.7 mm to -3.4 mm Bregma were collected. For lung samples, serial 4 μm-thick sections of the left lung were collected. Another subset of mice were euthanised by rapid decapitation following sodium pentobarbitone overdose, for collection of bronchoalveolar lavage fluid (BALF) via in situ lung lavage [45]. Mice were then decapitated, and the brains extracted from the skull. The cerebral cortices and hippocampi were dissected and rapidly snap-frozen in liquid nitrogen for gene expression analyses.

### Histology

To quantify tissue volume atrophy, six equidistant coronal brain Sect. (280 μm apart, approximately − 0.7 to -3.5 mm Bregma) were stained with Luxol Fast Blue (LFB) and Cresyl Violet (CV) as per a modified protocol [46]. For analysis, images were captured using a Leica Aperio AT Turbo Brightfield slide scanner, then FIJI/ImageJ (NIH, Bethesda, MD; http://imagej.nih.gov/ij) was used to apply the unbiased Cavalieri method to estimate the volume of the intact dorsal cortex and hippocampus in each hemisphere [47]. Each grid point represented an area of 100 μm², with a sampling frequency of four. Measurements were confined to the dorsal portion of each region of interest [48]. Group means were expressed as the ratio of ipsilateral to contralateral volumes.

Lung tissue sections were stained with hematoxylin and eosin (H&E) as per a standard protocol by the Monash Histology Platform, using the Leica ST5010 Autostainer XL (Leica Biosystems Imaging Inc.). Digital lung slides were viewed with Aperio ImageScope v.12.4.3 at 10x magnification. Alveolar airspace size was quantified using the mean linear intercept (MLI) method [45, 49]. Briefly, 20 equal length lines were drawn over images of H&E-stained lung cross-sections, then the number of intercepts with alveolar walls were counted. MLI was calculated by dividing the line length by the average intercepts per line. Analyses were performed on five regions of interest (ROIs) 500 × 500 μm in size selected randomly per lung section, and averaged across the top, middle and bottom sections of each lobe.

### Immunofluorescence staining

Brain sections were stained for astrocytic marker glial fibrillary acidic protein (GFAP) and neutrophil marker myeloperoxidase (MPO) to evaluate cellular immune responses. Heat-mediated antigen retrieval was performed using DAKO Target Antigen Retrieval Solution (diluted 1:10; Agilent Technologies, USA). After blocking with 10% normal donkey serum in 0.1% Triton-X100 in PBS, a rabbit polyclonal GFAP antibody (1:1000; Thermo Fisher Scientific) and goat polyclonal MPO (1:250; R&D Systems) were applied. Secondary antibodies used were donkey anti-rabbit AF 594, donkey anti-goat AF 488, or donkey anti-goat AF 594 (1:250; Thermo Fisher Scientific) and counterstained using Hoechst dye. Lung sections were stained with MPO as an indicator of neutrophil infiltration. Sections were dewaxed then rehydrated, followed by heat-mediated antigen retrieval with Tris-EDTA solution (pH 9.0). After blocking with CAS-Block™ Histochemical Reagent (Thermo Fisher Scientific, Waltham, MA, USA), sections were incubated overnight with a goat anti-mouse MPO antibody (1:50; R&D Systems, USA) diluted in 1% bovine serum albumin (BSA) in PBS. On day 2, a chicken anti-goat Alexa Fluor (AF) 594-conjugated secondary antibody was applied (1:200; Thermo Fisher Scientific), then counterstained with 4’,6-diamidino-2-phenylindole (DAPI).

Images were captured on a Nikon Eclipse Ti-E inverted microscope fitted with an Andor Zyla sCMOS camera and using the Nikon NIS-Elements v.5.30.06 software at 20x magnification. Stitched images of the entire lung section or ipsilateral dorsal hemisphere were captured and exported to FIJI ImageJ for manual cell counts (e.g., co-localized MPO/DAPI in the lung; MPO + cells in the injured brain) or determination of fluorescence percentage area (for GFAP), by a blinded investigator within specified ROIs (two for the cortex and one for the hippocampus), averaged across 2 sections per brain per marker.

### Cytokine analysis of BALF and serum

Cytokine and chemokine concentration was quantified in BALF and serum samples using a V-PLEX® Cytokine Panel 1 (mouse) and V-PLEX® Pro-inflammatory Panel 1 (mouse) (Meso Scale Diagnostics, LLC, Rockville, MD). These panels included detection of MCP-1 (CCL2), MIP-2 (CXCL2), IL-1β, IL-6, KC/GRO, IL-10, and TNFα. Detection was performed according to the manufacturer’s specifications on a MSD SQ120MM (VB204) instrument, with curves analyzed by Discovery Workbench® using a 4PL Curve fit for all analytes. Samples were assayed in duplicate by Crux Biolabs (Bayswater, VIC, Australia).

### Brain quantitative polymerase chain reaction (qPCR)

RNA was isolated from the ipsilateral (injured) cortex and hippocampus using the RNeasy® Mini Kit (Qiagen, Hilden, Germany). RNA purity was measuring using the QIAxpert spectrophotometer, then converted to cDNA using the QuantiTect® Reverse Transcription Kit (Qiagen). High throughput qPCR was performed by the Monash Health Translation Precinct (MHTP) Medical Genomics Facility using the Fluidigm BioMark HD™ (Standard BioTools Inc., San Francisco, CA, USA) in a Fluidigm 192.24 Dynamic Array™ Integrated Fluidic Circuit for Gene Expression. A panel of 24 genes were analyzed, including the housekeeping genes (HKGs) *Ywhaz* (Mm01722325_m1), *Ppia* (Mm02342430_g1) and *Hprt* (Mm03024075_m1) (Zamani et al., 2020), as well as the following cytokines, chemokines, immune cell markers, and immune-regulatory genes: *Il-1β* (Mm00434228_m1), *Il-1α* (Mm00439620_m1), *Tnf-α* (Mm99999068_m1), *Il-6* (Mm00446190_m1), *Il-10* (Mm01288386_m1), *Ccl2* (Mm00441242_m1), *Ccr2* (Mm00438270_m1), *Cxcl2* (Mm00436450_m1) and *Cxcr2* (Mm99999117_s1), *Cd45* (Mm01293577_m1) and *Cd86* (Mm00444543_m1), *Gfap* (Mm01253033_m1), *Aldh1l1* (Mm03048957_m1), *Arg1* (Mm00475988_m1), *Tmem119* (Mm00525305_m1), *Trem2* (Mm04209424_g1), *Nos2* (Mm00440485_m1), *Tgf1* (Mm01227699_m1), *Mapk1* (Mm00442479_m1), *Nfkb1* (Mm00476361_m1), and *Hmox1* (Mm00516005_m1). Relative gene expression was calculated using the 2^− ρρCT^ method and was normalized to the average expression of three reference genes.

### Fecal microbiome analysis

The fecal microbiome was sampled at five defined acute time points: (1) immediately prior to the TBI/sham procedure; (2) 24 h after the procedure; (3) on day 4 post-TBI/sham (prior to *K. pneumoniae*/vehicle inoculation); (4) 24 h post-inoculation with *K. pneumoniae*/vehicle; and (5) 7 d post-infection. Microbial DNA extraction and sequencing was performed using a modified published protocol [50, 51]. The QIAamp PowerFecal Pro DNA Kit (Qiagen, #51,084) was used to extract fecal DNA according to the manufacturer’s protocol. Three negative controls were included to address potential microbial DNA contamination at different steps of the procedure. For validation, positive controls were established by treating 5 µL of ZymoBIOMICS Microbial Community (Zymo Research) akin to the actual samples, successfully identifying 7 out of 8 bacterial species present in the positive control samples.

Each sample underwent PCR amplification using custom barcoded primers targeting the bacterial 16S rDNA v1-v2 region (F-27/R-338), with the following sequences: 16S-Forward: 5’-AATGATACGGCGACCACCGAGATCTACACTATGGTAATTCCAGMGTTYGATYMTGGCTCAG-3’; and 16S-Reverse: 5’-CAAGCAGAAGACGGCATACGAGATACGAGACTGATTNNNNNNNNNNNNAAGCTGCCTCCCGTAGGAGT-3’. Here, the ‘N’ sequences represented sample-specific 12-nucleotide Golay barcodes. Accuprime PCR buffer II and Accuprime Taq polymerase (Fisher Scientific) were used for PCR. Subsequent to quantification using a Fragment Analyzer (Agilent Technologies), amplicons were pooled at equimolar ratios, purified via the AMPure XP bead cleanup system (Beckman Coulter), and sequenced on a MiSeq platform using a MiSeq Reagent Kit v2.

### Microbial taxonomic profiling

Raw sequences were processed using the microbiome-dada2 pipeline (https://github.com/respiratory-immunology-lab/microbiome-dada2) using the DADA2 (version 1.28.0) R package [52]. Briefly, fastq files were demultiplexed using the iu-demultiplex function from illumina-utils tools [53], primers and adapters removed with cutadapt [54], reads filtered and trimmed, sequencing error models generated, sequences dereplicated, amplicon sequence variants (ASVs) inferred, paired-ends merged, and chimeras removed. Bacterial 16 S ASVs were assigned a taxonomy using the SILVA database train set (version 138) and the SILVA species assignment dataset (version 138) for exact sequence matching. A phylogenetic tree based on ASV sequences was built by performing a multiple-alignment using DECIPHER R package (version 2.28.0), followed by construction of a neighbor-joining tree as a starting point [55]. Contaminant taxa were identified using the isContaminant function of the decontam (version 1.20.0) R package [56], using DNA extraction and 16 S PCR water control samples as negative controls. The decontam ‘prevalence’ method was selected to identify contaminants as those that were more prevalent in negative controls than true samples (prevalence threshold = 0.5); contaminants were removed prior to filtering. Samples containing < 5,000 reads were excluded from the dataset and taxa present in < 5% of samples or lacking assignment at the phylum level were filtered out. Phylogeny-weighted Hill-Shannon diversity was calculated using the hill_phylo function from the hillR (version 0.5.2) R package [57]. ASV counts were normalised using Cumulative Sum Scaling (CSS) using the calcNormFactors function from the MetagenomeSeq (version 1.42.0) package [58], followed by log transformation.

### Statistical analysis

Most statistical analysis was performed using GraphPad Prism v.9.4.1 (GraphPad Software Inc., San Diego, CA, USA), with significance defined as *p* < 0.05. Two and three-way analyses of variance (ANOVA) were performed with Tukey’s post hoc test where appropriate. Data with 3 independent variables of time, injury, and infection, were assessed with a 3-way ANOVA. In most instances, both male and female mice are pooled per group, with open circle data points graphically denoting female animals. Potential sex differences were tested by 3-way ANOVA (factors of sex, injury, and infection), and only reported where significant sex differences were detected. Data are presented as mean ± SEM.

Microbiome data was analyzed using R (version 4.3.0). Differences in Hill-Shannon diversity were addressed using non-parametric Wilcoxon Rank Sum testing. Principle Coordinate Analysis (PCoA) was performed on the weighted UniFrac distance of normalized ASV counts using the ordinate function of the phyloseq (version 1.44.0) R package [59]. Permutational Multivariate Analysis of Variance (PERMANOVA) was subsequently performed on the same data with 1,000,000 permutations. Pre-treatment comparisons used the model ‘distance ∼ injury’, and post-treatment used ‘distance ∼ injury + treatment’. Longitudinal non-parametric repeated measures Brunner-Langer tests were performed to assess microbial differences due to injury and treatment using the f2.ld.f1 function from the nparLD (version 2.2) R package [60]. Linear modelling to assess timepoint-specific group-wise differences was performed using a custom wrapper around the limma R package [61] (https://github.com/mucosal-immunology-lab/microbiome-analysis/wiki/Limma-DA).

## Results

### Dose-dependent responses to *K. pneumoniae* in Naïve mice

We first conducted a series of pilot studies to determine the optimal dose of *K. pneumoniae* via intratracheal administration in naïve male and female C56Bl/6J mice (Fig. 1a) and evaluated changes in body weight (Fig. 1b), lung bacterial load (Fig. 1c) and mortality (Fig. 1d), alongside observable symptoms of illness. A low dose of 1 × 10^4^ CFU failed to induce any observable symptoms in inoculated mice, and a negligible bacterial load in the lungs at 24 h (< 3 CFU/lung; *n* = 3; not shown). Mice that received a dose of 1 × 10^5^ CFU had a low bacterial load, and exhibited only minimal symptoms (inactive). The intermediate dose of 1 × 10^6^ CFU induced overt clinical symptoms of illness acutely (e.g., inactive, fur erect, hunched posture, acute dyspnea), and an intermediate lung bacterial load. Mice that received the highest dose of 1 × 10^7^ CFU were found to have a high lung bacterial load, exhibited pronounced weight loss within 48 h, and presented with significant clinical symptoms of acute respiratory illness. This dose also resulted in considerable mortality (63%; *n* = 5/8) within the first week post-infection (2/4 males and 3/4 females), between 2 and 3 days post-inoculation, as animals reached the weight loss threshold requiring humane euthanasia. No mortality was observed for mice that received a dose of 1 × 10^6^ CFU *K. pneumoniae* or vehicle (Fig. 1b).

In a separate pilot experiment, we compared intratracheal treatments of sterile saline only versus broth (the vehicle for *K. pneumoniae* inoculation), and found no mortality, comparable body weights, no signs of acute illness, and no differences in lung histological measures (*n* = 4/group; not shown). Subsequent experiments therefore employed a broth solution vehicle or a dose of 1 × 10^6^ CFU *K. pneumoniae*.

### *K. pneumoniae* administration after TBI does not worsen acute symptoms of illness

**Fig. 2:**
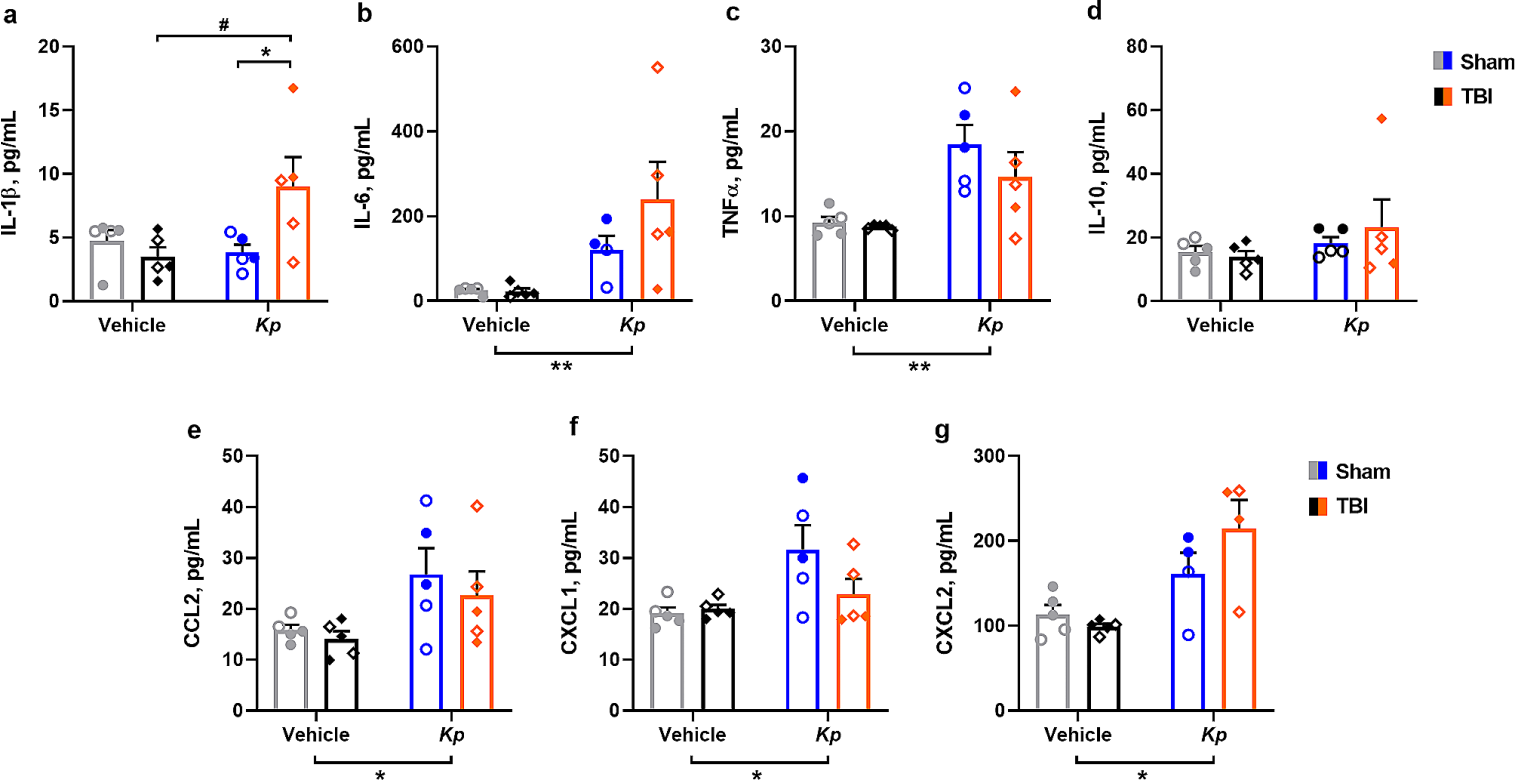
*K. pneumoniae* infection causes transient hypoactivity and weight loss in both TBI and Sham mice. The experimental timeline (**a**), whereby TBI or sham mice were inoculated with *Kp* or vehicle after 4 days, then were assessed repeatedly at 4 h, 24 h and 7 d post-infection (OF = Open Field behavior test). At 24 h post-infection, the lung bacterial load was equivalent in sham and TBI mice (**b**). Changes in body weight across the 7 d time course (**c**; *n* = 10/group) revealed a main effect of time, and a time-x-*Kp* interaction (*p < 0.05 from post-hoc analyses, 3-way RM ANOVA). In the OF test at 4 h post-infection, a non-significant trend towards reduced time in the center was observed in *Kp*-treated mice (**d**), which was resolved by 24 h and 7 d (not shown). Also in the OF test, TBI induced an increase in activity at 4 h, 24 h and 7 d (e-g; 2-way ANOVAs, main effect of TBI *p < 0.05, ***p < 0.001), while *Kp* independently reduced activity just at 4 h post-infection (**e**; ****p < 0.0001). n’s in (**d**–**g**) are depicted by individual data points; males indicated by closed circles/diamonds and females indicated by open circles/diamonds

To assess whether *K. pneumoniae* infection after TBI altered functional outcomes, mice were subjected to TBI or sham surgery, followed by *K. pneumoniae* or vehicle inoculation at 4 days post-injury, and examined over the subsequent 7 d period (Fig. 2a). The lung bacterial load at 24 h post-infection was comparable between Sham-*Kp* and TBI-*Kp* mice (t_8_ = 0.62, *p* = 0.5529; Fig. 2b), and undetectable in vehicle-treated mice. Spleens were collected fresh and weighed at either 24 h or 7 d post-infection, as a surrogate indication of mobilization of the peripheral immune response. However, neither TBI nor *K. pneumoniae* altered spleen weight (either raw or relative to body weight) (not shown; *p* > 0.05).

Body weights were assessed daily as an indicator of general health. A main effect of time was observed (F_11, 396_=21.30, *p* < 0.0001), as well as a time-x-*Kp* interaction (F_11, 396_=3.75, *p* < 0.0001). All groups appeared to exhibit comparable mild weight loss (approximately 2–4%) in the first 24 h post-TBI/sham surgery then quickly regained it, although this change was not statistically significant. Then, at 24 h (1 day) post-inoculation, both sham-*Kp* and TBI-*Kp* groups showed a similar reduction in body weight compared to vehicle-treated groups (post-hoc *p < 0.01 compared to Sham-Vehicle); although again this was only a transient drop. When cohorts were separated by sex (*n* = 3/group; Supplementary Fig. 1), it was noted that males showed more consistent body weights across the time course; whereas females showed considerable within-group variability. The effect of *Kp* on body weight was more pronounced in female mice, as a time-x-*Kp* interaction was detected in females (F_11, 176_=2.39, *p* = 0.0087) but not males (F_11, 176_=1.53, *p* = 0.1244).

The open field test was conducted to evaluate locomotor activity and anxiety-like behavior. At 4 h post-infection, a non-significant trend was seen with *K. pneumoniae*-treated mice appearing to spend less time in the center of the arena (F_1, 35_=3.72, *p* = 0.0619), suggestive of increased anxiety-like behavior (Fig. 2d). At the same time, independent main effects of both TBI (F_1, 35_=61.62, *p* = 0.0255) and *K. pneumoniae* (F_1, 35_=5.45, *p* < 0.0001) were observed for distance traveled in the open field, with TBI mice moving more but *K. pneumoniae*-treated mice moving less (Fig. 2e). This hypoactive effect of *K. pneumoniae* was transient, as it had resolved by the 24 h (Fig. 2f) and 7 d (Fig. 2g) time points. However, a main effect of TBI was observed across the time course (F_1, 59_=6.38, *p* = 0.0143 at 24 h, and F_1, 36_=13.67, *p* = 0.0007 at 7 d).

### Elevated serum inflammatory cytokines in response to *K. pneumoniae*

**Fig. 3:**
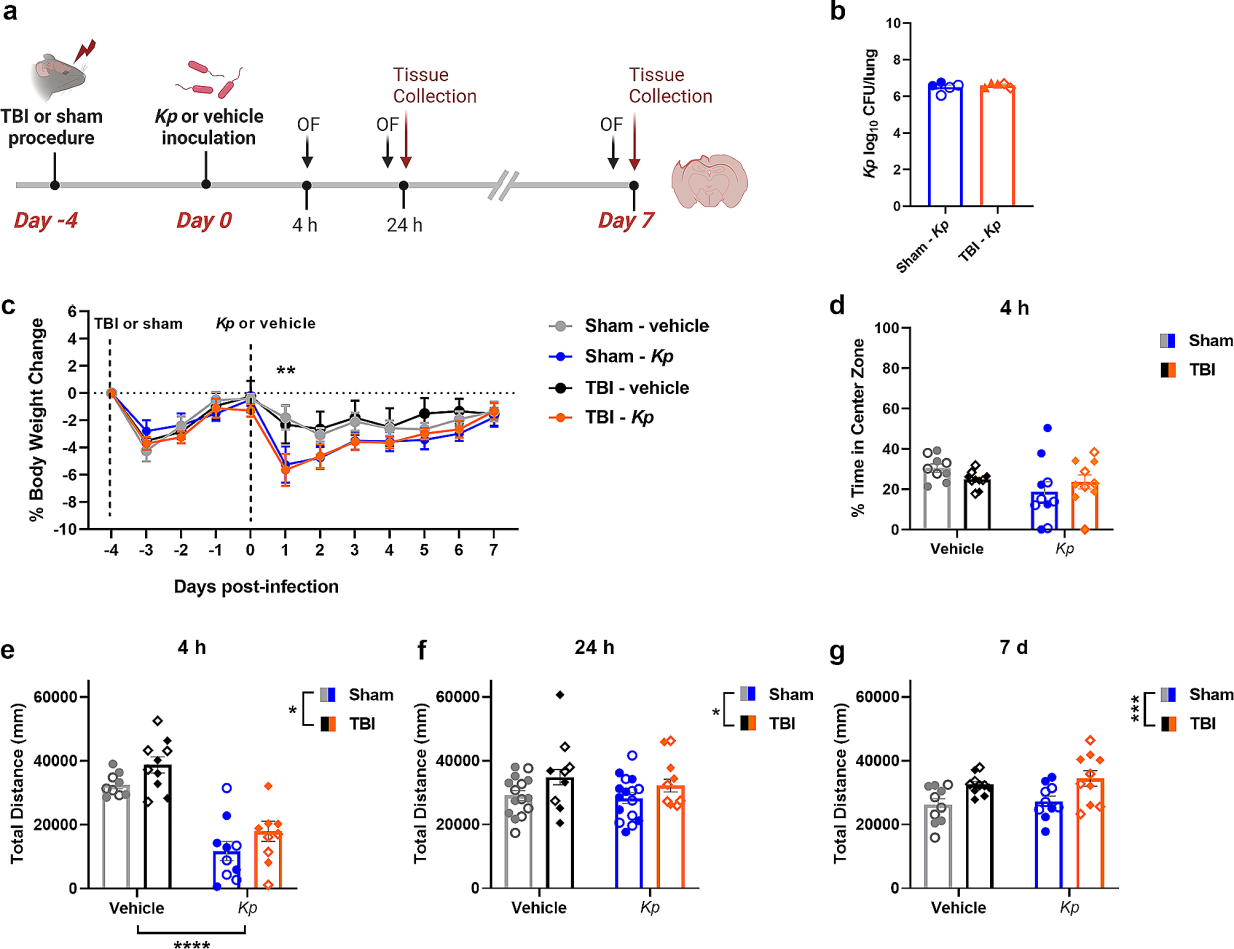
Elevated Serum Cytokines at 24 h Post-Infection. Cytokine levels in serum at 24 h post-infection (**a**–**g**), reveal a pro-inflammatory effect of *K. pneumoniae*, but no influence of a prior TBI on this response (2-way ANOVAs, main effects of Kp *p < 0.05, **p < 0.01). In (**a**), *p < 0.05 indicates Tukey’s post-hoc comparison (TBI-vehicle vs. TBI-*Kp*) following a significant TBI-x-*Kp* interaction. Males denoted by closed circles/diamonds and females by open circles/diamonds. *n* = 4–5/group

For prototypical pro-inflammatory cytokine IL-1β (Fig. 3a), a significant TBI-x-*Kp* interaction was detected (F_1, 16_=5.95, *p* = 0.0267), whereby IL-1β was elevated in the serum of TBI-*Kp* mice compared to TBI-vehicle (Tukey’s post-hoc, *p* = 0.0397). Independent of TBI, *K. pneumoniae* induced a robust increase in cytokines IL-6 (Fig. 3b; F_1, 15_=10.01, *p* = 0.0064) and TNFα (Fig. 3c; F_1, 15_=15.86, *p* = 0.0011), as well as the chemokines CCL2 (Fig. 3e; F_1, 16_=7.15, *p* = 0.0167), CXCL1 (Fig. 3f; F_1, 15_=7.21, *p* = 0.0163) and CXCL2 (Fig. 3g; F_1, 16_=17.29, *p* = 0.0010). By contrast, levels of anti-inflammatory cytokine IL-10 were not altered by *K. pneumoniae* or TBI (Fig. 3d).

### Lung inflammation and pathology after *K. pneumoniae* infection

**Fig. 4:**
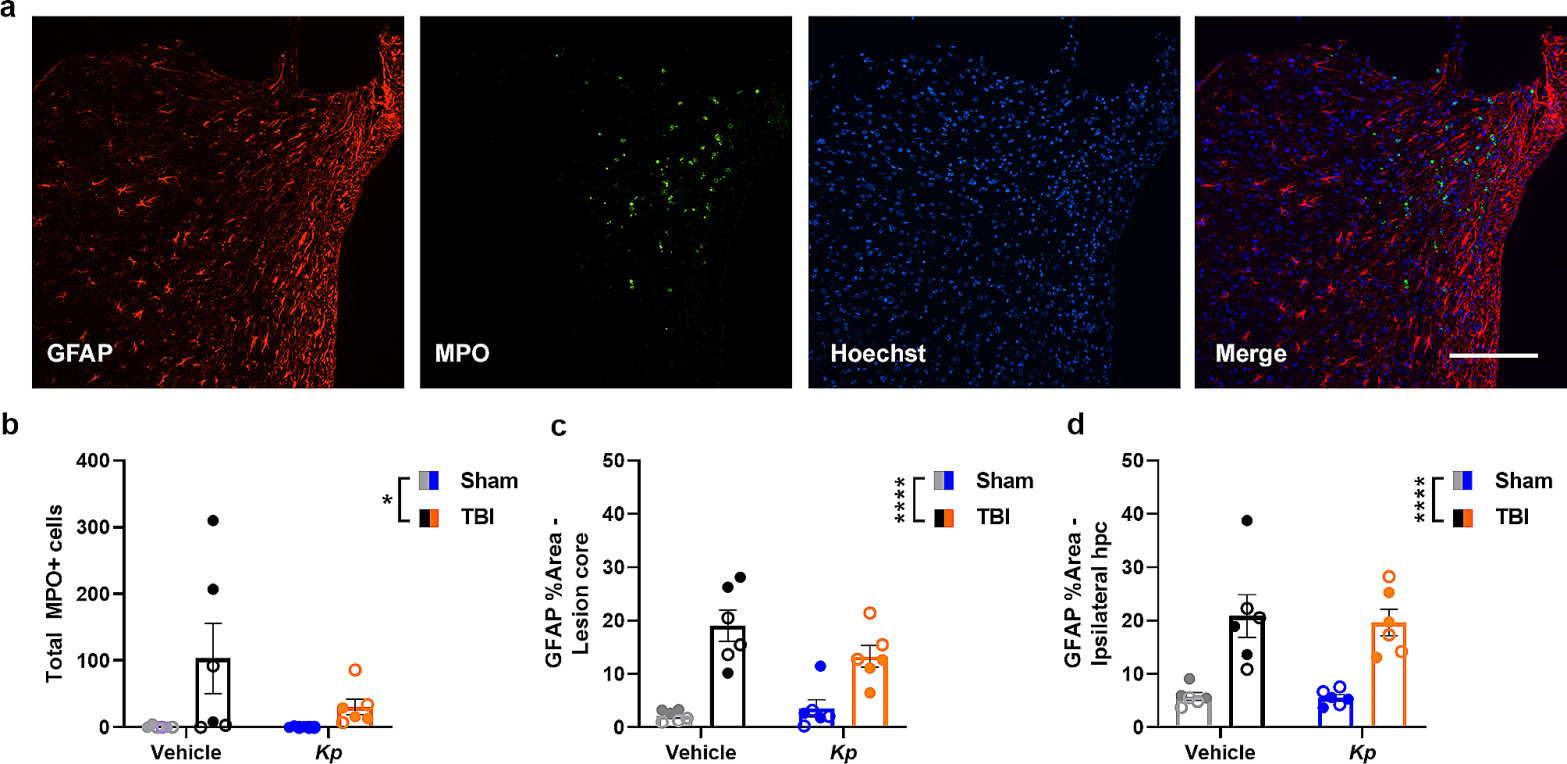
*K. pneumoniae* Infection Alters Lung Histology and BALF Cytokine Levels. Cytokine levels in bronchoalveolar fluid (BALF) at 24 h post-infection (**a**–**f**, **h**) reveal a predominantly pro-inflammatory effect of *K. pneumoniae* but no effect of prior TBI (2-way ANOVAs, main effect of *Kp*; *p < 0.05; **p < 0.01; ****p < 0.0001). Immunostaining for MPO + neutrophils (colocalized with nuclear marker DAPI; **g**) in lung tissue revealed a robust neutrophilic infiltration at 24 h post-infection, particularly associated with vascular thrombi (*). By 7 d post-infection, this immune response had resolved, with no differences in MPO + cell numbers between the groups (**i**). Representative H&E staining illustrates lung histology at 7 d post-infection (**j**). Quantification of the mean linear intercept (MLI) (**k**), where an increase reflects pathology, revealed an increase in lungs from TBI-*Kp* mice (2-way ANOVA, TBI-x-*Kp* interaction; Tukey’s post-hoc *p < 0.05). Males denoted by closed circles/diamonds and females by open circles/diamonds. *n* = 4–6/group. Scale bar = 50 μm (**g**) and 100 μm (**j**)

Pulmonary inflammation and lung pathology was evaluated in a subset of animals at 24 h and 7 d post-*K. pneumoniae* infection, to evaluate whether the combined insult of TBI plus *K. pneumoniae* worsened pulmonary outcomes. At 24 h post-infection, when the presence of bacteria was confirmed in lung homogenates (Fig. 2b), the analysis of inflammatory cytokines in BALF revealed a robust pro-inflammatory response in *K. pneumoniae*-treated mice compared to vehicle. Specifically, *K. pneumoniae* induced a robust increase in cytokines IL-1β (Fig. 4a; F_1, 15_=4.66, *p* = 0.0476), IL-6 (Fig. 4b; F_1, 15_=27.99, *p* = 0.0001), TNFα (Fig. 4d; F_1, 15_=27.82, *p* < 0.0001) and IL-10 (Fig. 4e), as well as the chemokines CCL2 (Fig. 4c; F_1, 15_=13.43, *p* = 0.0023), CXCL2 (Fig. 4f; F_1, 15_=13.66, *p* = 0.0022) and CXCL1 (Fig. 4h; F_1, 16_=12.53, *p* = 0.0027). However, a TBI sustained 4 days prior to *K. pneumoniae* infection did not alter BALF cytokine levels.

Significant correlations between serum and BALF levels of several cytokines were noted, for IL-1β (r^2^ = 0.50, *p* = 0.0007), TNFα (r^2^ = 0.32, *p* = 0.0121), IL-6 (r^2^ = 0.43, *p* = 0.0024), CXCL1 (r^2^ = 0.63, *p* < 0.0001), CXCL2 (r^2^ = 0.25, *p* = 0.0256) and IL-10 (r^2^ = 0.38, *p* = 0.0047), and a trend observed for CCL2 (r^2^ = 0.20, *p* = 0.0559), suggesting that the local pulmonary response was the source of inflammatory mediators in the serum. Considerable neutrophil infiltration was observed by MPO immunofluorescence staining of lung tissue at 24 h, particularly associated with vascular thrombosis (Fig. 4g). Qualitatively this infiltration appeared comparable between Sham-*Kp* and TBI-*Kp* groups, although quantification was not readily possible due to the dense clustering of these cells at this time.

By 7 d post-infection, fewer MPO + cells were detected in lung tissue (Fig. 4i) and quantification revealed similar numbers across all groups (n.s.). Also at 7 d post-infection, calculation of the mean linear intercept (MLI) to evaluate lung pathology from H&E-stained sections (Fig. 4j) revealed a significant TBI-x-*Kp* interaction (F_1, 20_=5.56, *p* = 0.0287) and a trend towards a main effect of *K. pneumoniae* (F_1, 20_=3.71, *p* = 0.0685). From Tukey’s post-hoc comparisons, *K. pneumoniae* increased the MLI in TBI mice specifically (TBI-vehicle vs. TBI-*Kp*; *p* < 0.05), indicating the presence of pathology (reduced structural integrity of the alveolar septum/increased alveolar size). The TBI-*Kp* group trended towards a higher MLI compared to Sham-*Kp* mice (*p* = 0.0593; Fig. 4k).

### Immune-related gene expression changes in the injured brain after *k. pneumoniae* infection

**Fig. 5:**
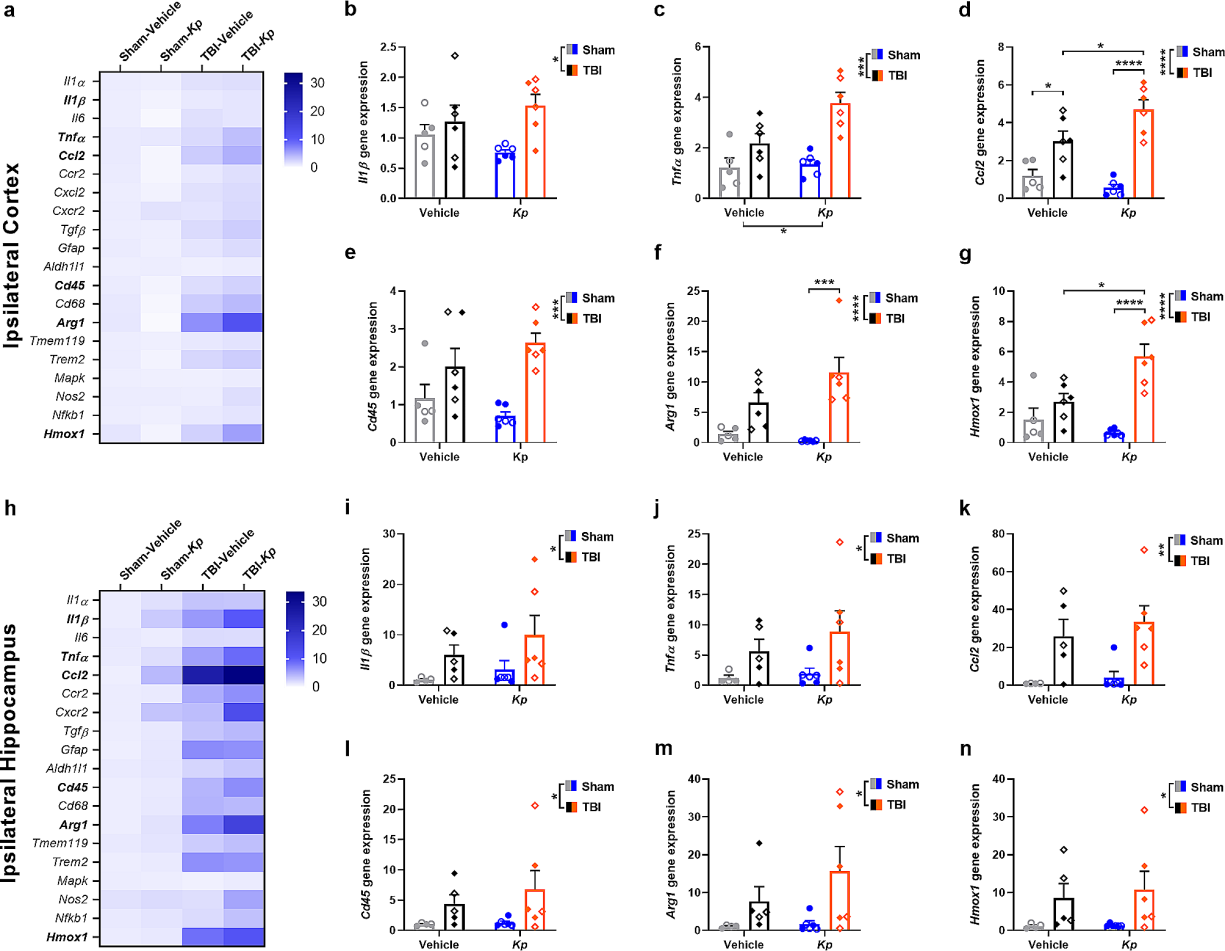
*K. pneumoniae (Kp)* infection had a minimal impact on immune-related gene expression in the injured brain. Quantatitive PCR evaluated the relative expression of multiple genes of interest, with group means depicted in heatmaps of the ipsilateral cortex (**a**) and ipsilateral hippocampus (**h**) at 24 h post-*Kp*/vehicle (= 5 d post-TBI/sham). Heatmap key illustrates that increasing color saturation reflects higher gene expression relative to the Sham-vehicle control group (bolded gene names are those that are also depicted graphically; scale = fold change gene expression vs. Sham-Vehicle). Group comparisions for select genes of interest are shown for the cortex (**b**–**g**) and hippocampus (**i**–**n**), with analysis by 2-way ANOVAs followed by Tukey’s post-hoc comparisons when a significant TBI-x-*Kp* interaction was observed (i.e., for **d**, **g** and **f**). Males are indicated by closed circles/diamonds and females by open circles/diamonds. *n* = 4–6/group

We next considered the potential effect of *K. pneumoniae* lung infection on the brain’s immune response to TBI, by examining the expression profiles of key genes involved in regulating neuroinflammation–including cytokines, chemokines, immune cell markers, and downstream signaling mediators. Broadly, at 24 h post- *K. pneumoniae* infection (= 5 d post-TBI/sham), most genes exhibited upregulation in response to TBI in the ipsilateral cortex (Fig. 5a) and ipsilateral hippocampus (Fig. 5h). In the cortex, TBI induced increased expression of cytokines *Il1β* (Fig. 5b; F_1, 19_=6.91, *p* = 0.0165) and *Tnfα* (Fig. 5c; F_1, 19_=23.03, *p* = 0.0001), chemokine *Ccl2* (Fig. 5d; F_1, 19_=47.93, *p* < 0.0001), leukocyte marker *Cd45* (Fig. 5e; F_1, 19_=17.54, *p* = 0.0005), as well as immune mediators *Arg1* (Fig. 5f; F_1, 19_=27.83, *p* < 0.0001) and *Hmox1* (Fig. 5g; F_1, 19_=26.20, *p* < 0.0001). Independently, *K. pneumoniae* induced an increase in *Tnfα* only (F_1, 19_=5.96, *p* = 0.0246). However, a significant TBI-x-*Kp* interaction was observed for *Ccl2* (F_1, 19_=7.06, *p* = 0.0156) and *Hmox1* (F_1, 19_=9.99, *p* = 0.0051), as well as a non-significant trend towards an interaction for *Arg1* expression (F_1, 19_=3.73, *p* = 0.0685). From Tukey’s post-hoc analyses, TBI-*Kp* mice were found to have higher levels of *Ccl2* expression compared to either TBI-vehicle or Sham-*Kp* mice (Fig. 5d; *p* < 0.05). *Arg1* expression was only elevated in TBI-*Kp* compared to Sham-*Kp* mice (Fig. 5f); while for *Hmox1*, expression in TBI-*Kp* mice was higher compared to both TBI-Vehicle and Sham-*Kp* mice (Fig. 5g). Altogether, TBI induced a robust pro-inflammatory immune state in the injured cortex, and post-injury *K. pneumoniae* lung infection appeared to potentiate this response for select genes.

In the hippocampus (Fig. 5i-n), examination of the same key genes revealed main effects of TBI (*Il1β*: F_1, 17_=4.91, *p* = 0.0406; *Tnfα*: F_1, 17_=5.86, *p* = 0.0270; *Ccl2*: F_1, 17_=15.63, *p* = 0.0010; *Cd45*: F_1, 17_=5.00, *p* = 0.0391; *Arg1*: F_1, 17_=5.76, *p* = 0.0281; *Hmox1*: F_1, 17_=6.21, *p* = 0.0233). However, no independent effects of *K. pneumoniae* were observed, nor were there any TBI-x-*Kp* interactions. The chemokine *Cxcl2* was minimally detected in the cortex, but below the threshold for detection in the hippocampus, while the anti-inflammatory cytokine *Il-10* was below detection in both brain regions regardless of experimental group.

### Cellular neuroinflammation after TBI was not affected by *K. pneumoniae* infection

**Fig. 6:**
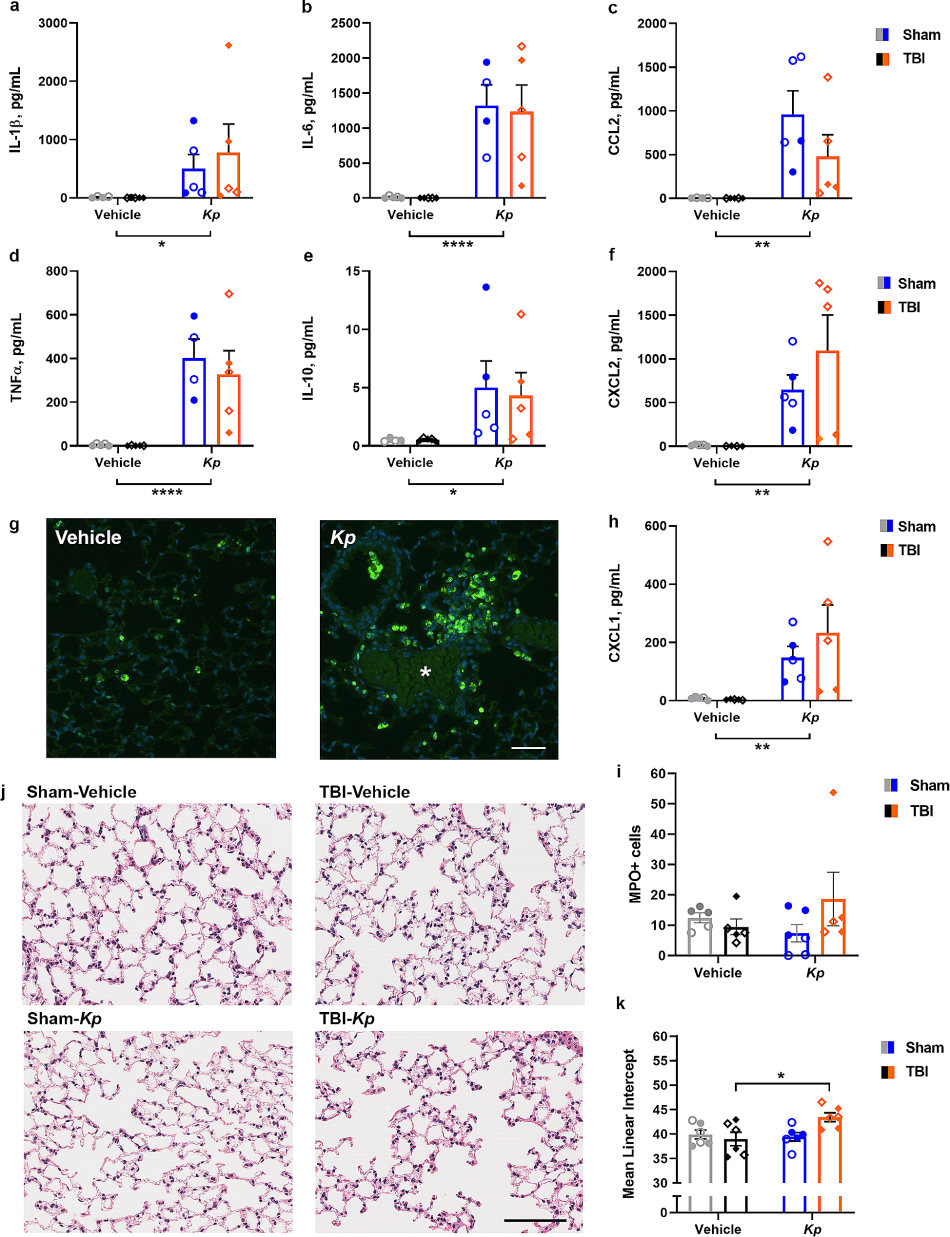
Immunofluorescence staining of cellular neuroinflammation after TBI and *K. pneumoniae* infection. Representative immunofluorescence images from the ipsilateral (injured) cortex of a TBI-Vehicle mouse at 11 days post-injury (7 d post-vehicle), illustrating detection of GFAP + astrocytes and MPO + neutrophils (**a**). Scale bar = 100 μm. Quantification of the total number of MPO + cells in the injured hemisphere (**b**), as well as GFAP % area coverage in the lesioned cortex (**c**) and hippocampus (**d**), revealed a main effect of TBI but no effects of *K. pneumoniae* (2-way ANOVA, *p < 0.05, ****p < 0.0001)

At a sub-acute time point of 7 d post-infection (= 11 d post-injury), markers of inflammatory immune cells were detected by immunofluorescence staining on coronal sections to evaluate whether *K. pneumoniae* altered their infiltration or activation (Fig. 6a). Quantification of MPO + cells, presumably neutrophils, revealed a TBI-induced increase (Figs. 2 and 6a-way ANOVA F_1, 20_ = 6.04, *p* = 0.0232). This response was highly variable, ranging from 0 to 310 in TBI brains. Quantification of GFAP + astrogliosis was performed by determining the proportion of GFAP + coverage of a consistent region-of-interest positioned in the core of the cortical lesion (Fig. 6c) or dorsal hippocampus (Fig. 6d). TBI induced a robust increase in GFAP reactivity at this time in both regions of ∼ 20% compared to ∼ 5% in sham-operated mice (F_1, 20_ =45.63, *p* < 0.0001; F_1, 20_ = 37.22, *p* < 0.0001), to a similar extent in both vehicle- and *K. pneumoniae*-treated mice.

### The extent of brain tissue damage after TBI was not affected by *K. pneumoniae*

**Fig. 7:**
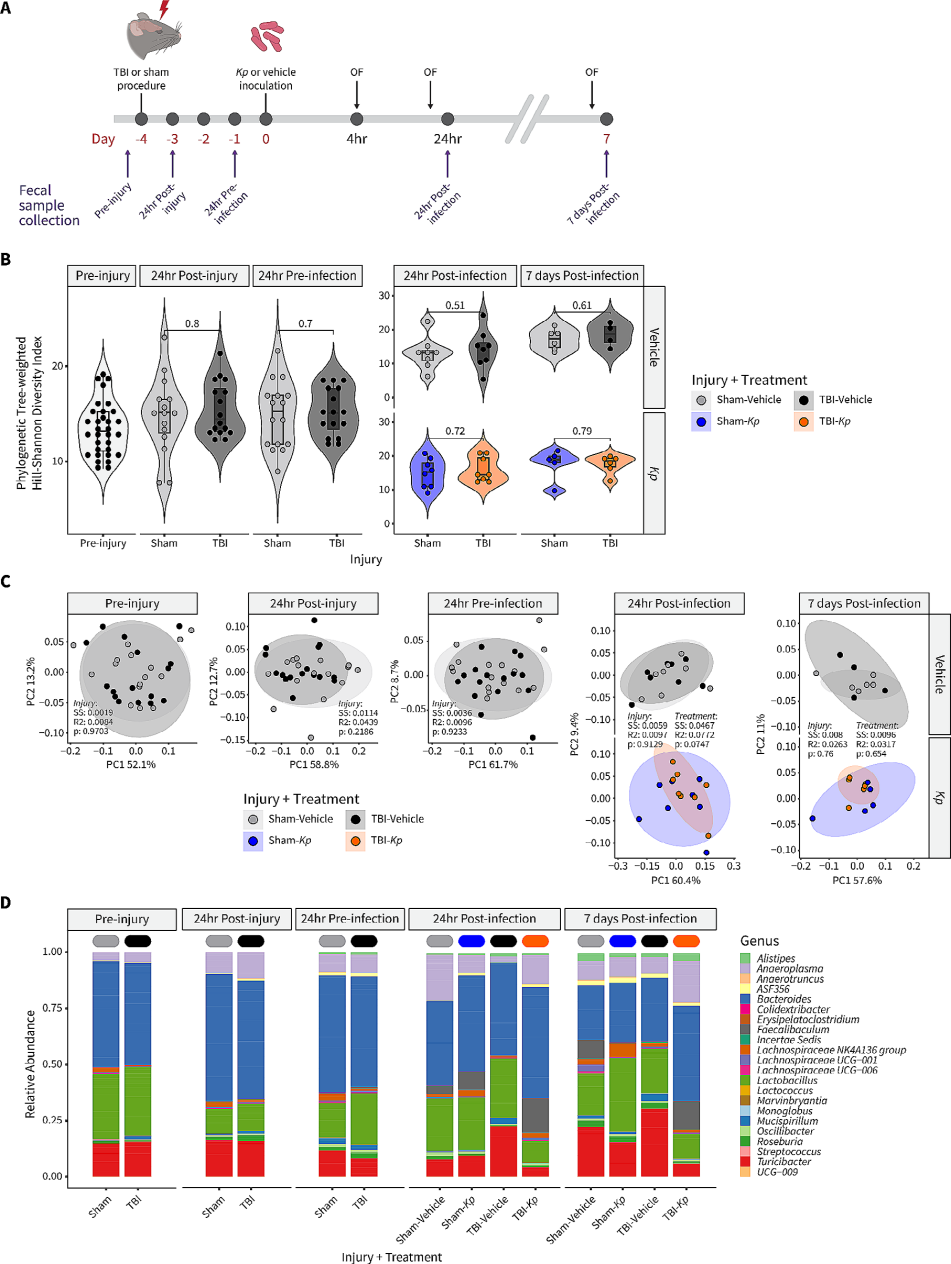
*K. pneumoniae* Infection Did Not Alter Cortical and Hippocampal Neuropathology After TBI. Representative brain sections from each experimental group (**a**) illustrate the extent of tissue damage from CV/LFB staining. Quantification of remaining intact tissue in the cortex (**b**) and hippocampus (**c**), expressed as ipsilateral relative to contralateral regions, demonstrates the considerable loss of tissue as a result of TBI (2-way ANOVA, **p < 0.01; ****p < 0.0001), but no effect of *K. pneumoniae* nor a TBI-x-*Kp* interaction. Males are indicated by closed circles and females by open circles. Scale bar = 1000 μm

At 7 days post-infection (= 11 days post-TBI), brains were collected, sectioned, and stained with CV and LFB to allow for histopathological assessment (Fig. 7a). Volumetric analysis of the remaining ipsilateral cortex tissue revealed a significant effect of TBI on cortex volume (2-way ANOVA F_1, 20_=97.85, *p* < 0.0001; Fig. 7b), as well as loss of hippocampal tissue compared to sham controls (F_1, 20_=10.55, *p* = 0.004; Fig. 7c). However, *K. pneumoniae* and vehicle-treated mice did not differ. Consideration of sex as a variable by 3-way ANOVA revealed a main effect of sex on cortical volume only (F_1, 16_=6.70, *p* = 0.0198), but no interactions, indicating that females had a slightly larger ipsilateral : contralateral cortex volume overall.

### Fecal bacterial diversity and composition were not altered by TBI or lung infection

The fecal microbiome was examined, across a time course post-injury and *K. pneumoniae* infection, as recent evidence supports a complex bi-directional relationship between the brain and the gut in the context of neurotrauma as well as in infection-related immune responses [38–40].

**Fig. 8:**
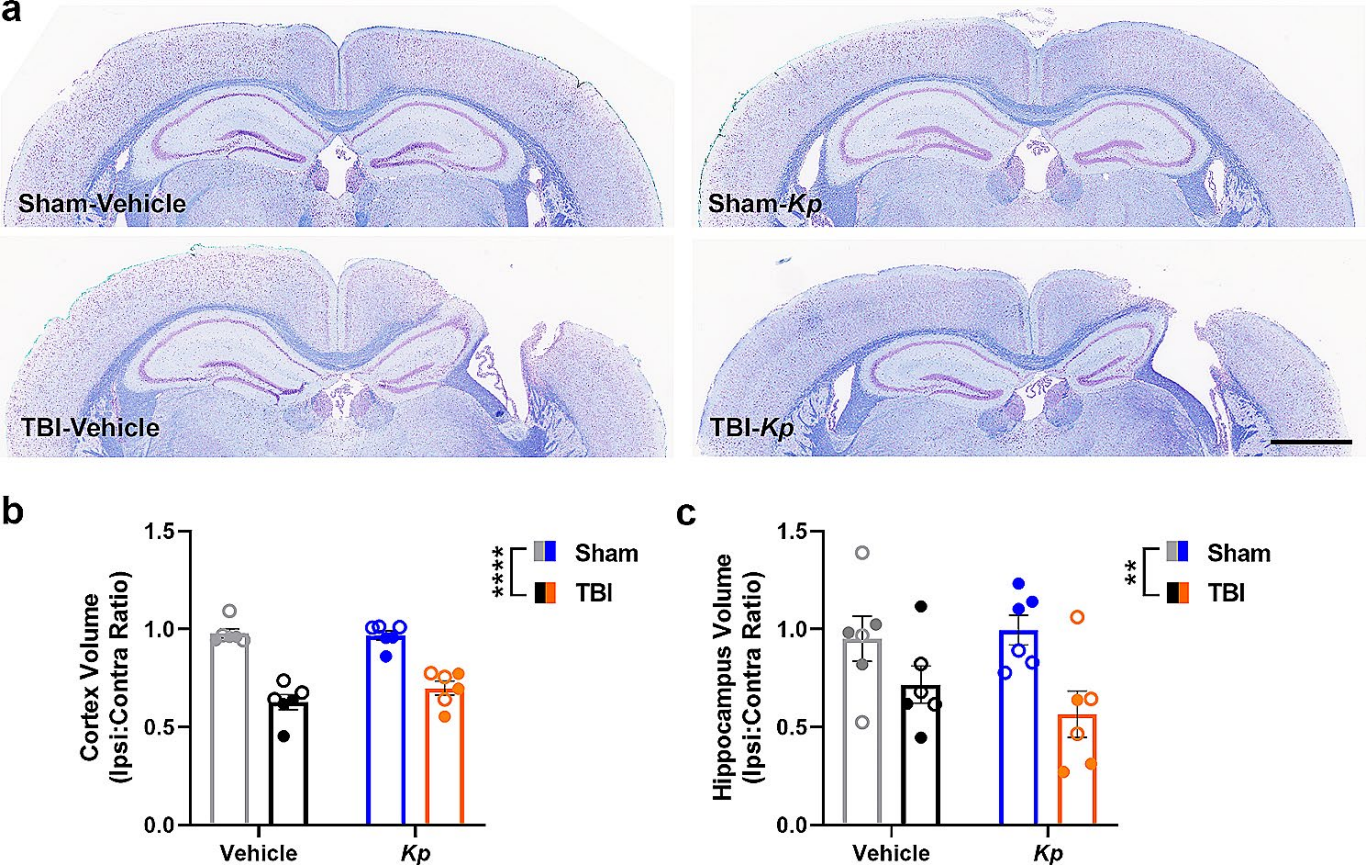
The Fecal Microbiome was Not Altered by TBI or Lung Infection. (**a**) Time line of sample collection. (**b**) Violin plots representing phylogenetic tree-weighted Hill-Shannon alpha-diversity. (**c**) Principle coordinate analysis (PCoA) plots of the weighted UniFrac distances shown in the first two principal coordinates for bacterial taxonomic composition. Ellipses represent the 90% confidence interval around the group centroid. Results for PERMANOVA testing are shown for injury and treatment differences (SS [sum of squares] shows effect size and R2 shows variance explained). (**d**) Bacterial genus-level relative abundance data grouped by injury + treatment group and timepoint

Alpha-diversity, as measured using the phylogenetic tree-weighted Hill-Shannon metric, was comparable between injury and treatment groups at all time points (Fig. 8B). Likewise, beta-diversity was similar between groups, and PERMANOVA tests to assess the impact of injury and *K. pneumoniae* on community structure revealed no significant differences at any time point (Fig. 8C). Fecal samples at all time points showed high abundance of *Bacteroides*, *Roseburia*, and *Turicibacter* (Fig. 8D).

At both the ASV and genus level, non-parametric repeated-measures Brunner-Langer tests did not detect any effect of injury, *K. pneumoniae*, or time on bacterial abundance. Subsequent linear modelling approaches at individual time points to investigate potential group-wise effects also showed no significant differences between groups.

## Discussion

The risk of contracting ventilator-associated pneumonia is particularly high for hospitalized patients with severe TBI [17]. As an opportunistic bacterium of the *Enterobacteriaceae* family, *K. pneumoniae* is a leading cause of ventilator-acquired pneumonia, urinary tract infections, and sepsis in immunocompromised and critically-ill individuals, including patients with brain injuries [19, 21, 62]. Indeed, recent studies of TBI patients in Taiwan and Saudi Arabia reported *K. pneumoniae* to be among the most prevalent pathogen in pulmonary infections [20, 63]. There is thus a clear need to better understand the consequences of *K. pneumoniae* infection in the context of TBI. In this study, we sought to establish and characterize a new mouse model of experimental TBI combined with a pulmonary *K. pneumoniae* infection, and evaluate the consequences of this combined insult on acute and sub-acute outcomes.

In pilot studies, we first sought to optimize the dose of *K. pneumoniae* required to yield a detectable bacterial burden in the lungs alongside relevant symptomology, while keeping mortality of experimental animals low. Although intratracheal inoculation requires anesthesia, an additional burden on the animals, we chose this delivery method as it is typically more reproducible than what can be achieved via inhalation [31]. A dose of 1 × 10^4^ or 1 × 10^5^ CFU had minimal effect on the mice, with few symptoms, negligible body weight loss, and no mortality. Conversely, a dose of 1 × 10^7^ CFU led to considerable mortality (> 50%) and significant weight loss (≥ 15% in survivors), at a time course consistent with what has been reported previously [64]. An intermediate dose of 1 × 10^6^ CFU was therefore chosen, as it led to moderate body weight loss at 1–2 days post-inoculation, but no mortality. The presence of *K. pneumoniae* in the lungs was confirmed at this time, but largely undetectable by the 7 day time point. This was consistent with previous reports that live *K. pneumoniae* rapidly expands in the lungs within the first 24 h before rapid clearance over the first week [35–37], a process that is largely neutrophil-mediated [65].

Next, we performed experimental TBI and infected mice with 1 × 10^6^ CFU *K. pneumoniae* at 4 days post-injury, attempting to recapitulate the time frame when TBI patients are most vulnerable and exposed to hospital-acquired infections. *K. pneumoniae* induced significant transient weight loss to a similar extent in both Sham-*Kp* and TBI-*Kp* groups, with a comparable trajectory of recovery. Infected mice showed sickness behaviors including a reduction in general activity at 4 h post-injury, but this was resolved by 24 h and 7 days post-infection; while TBI independently induced a modest increase in activity across the time course, consistent with previous findings in the CCI mouse model [41, 66]. Peripherally, spleen weights were unaffected by either TBI or *K. pneumoniae*, in contrast to a previous study that reported splenic thrombi and spleen enlargement due to expansion of myeloid precursors in mice exposure to lung K. pneumoniae infection [67].

However, serum cytokine levels measured at 24 h after *K. pneumoniae* inoculation reflected activation of the innate immune response, with an increase in pro-inflammatory cytokines IL-6 and TNFα, as well as prototypical chemokines CCL2, CXCL1 and CXCL2. IL-1β was elevated specifically in the TBI-*Kp* group, suggestive of an additive effect of TBI and infection for this mediator. Of note, TBI alone did not appear to increase most of the measured cytokines, although this was likely due to the time point chosen (past the peak of TBI-induced cytokine release). One limitation here is that the bacterial load in blood was not quantified, although the transient time course of symptoms in infected animals suggests that septicemia was unlikely to be occurring.

In the infected lungs, analysis of BALF revealed a robust pro-inflammatory response associated with *K. pneumoniae* infection, of a much higher magnitude than what was observed in the serum. This finding is consistent with evidence that *K. pneumoniae* induces extensive inflammation of the lungs [65, 68, 69], and our immunostaining to detect marked neutrophilic pulmonary infiltration acutely (although quantification of this at an acute time point was not possible in this study due to a technical issue). With an increasing awareness of brain-lung interactions and the potential for an altered immune response as a consequence of brain injury [as reviewed elsewhere; e.g., 70], we had hypothesized that TBI alone may influence lung immune responses. For example, in models of lung infections with *Pseudomonas aeruginosa*, some studies have observed reduced bacterial clearance in animals that had previously sustained a TBI [32], while others have seen a reduced bacterial burden (presumably increased bacterial clearance) in TBI mice post-infection [71–73]. However, this was not the case in our model. No additive effects were associated with the prior TBI in TBI-*Kp* mice, nor was there any difference in the bacterial load in the lungs of Sham-*Kp* versus TBI-*Kp* mice. Our findings align with those of Vermeij and colleagues, who evaluated TBI in adult rats followed by intratracheal administration of heat-killed *Staphylococcus aureus* and reported that the severity of pneumonia was not different in TBI compared to sham rats, despite evidence of TBI alone resulting in significant pulmonary edema and systemic immunosuppression [34]. Taken together, evidence suggests that the bacterial load, species, strain and its virulence are all likely to be important determinants of the effects of experimental infection on the lungs’ immune response, in addition to other insults (e.g., a prior TBI) and its relative timing and severity.

By 7 days, when published evidence [64] and our pilot data indicated that most of the bacteria had been cleared (e.g., few if any bacteria detectable, and MPO + cells at baseline levels), a modest level of lung pathology was evident from the analysis of H&E staining in the TBI-*Kp* group specifically, compared to either TBI or *K. pneumoniae* alone. One caveat is that we did not measure the bacterial load at the 7 d time point, so cannot conclusively state that the time course to resolution of *K. pneuomiae* infection was comparable in Sham-*Kp* versus TBI-*Kp* mice. However, together, these findings indicate that the moderate dose of *K. pneumoniae* used in this study induced a transient pulmonary infection that is well-countered by the inflammatory immune response. By contrast, severe *K. pneumoniae* associated with a higher bacterial load or more virulent strain can induce severe bacterial pneumonia, resulting in hemorrhage and the formation of necrotic lesions, and progression to bacteremia and sepsis [69]. However, such pathology is typically associated with significant mortality, which would be incompatible with an ethically-acceptable animal model – particularly when combined with the additional insult of a TBI.

In the injured brain, we hypothesized that a post-injury *K. pneumoniae* infection would exacerbate neuroinflammation and neuropathology. Focusing on the injured cortex and hippocampus at 24 h after *K. pneumoniae* infection, we found that many genes involved in regulating neuroinflammation—including cytokines, chemokines, immune cell markers, and downstream signaling mediators—were upregulated in response to TBI. However, *K. pneumoniae* had only minimal effects on gene expression. For example, in the injured cortex, *K. pneumoniae* independently increased *Tnfα* expression; and an interaction between TBI and *K. pneumoniae* was noted for *Ccl2*, *Hmox1* and potentially *Arg1*, whereby the combination of TBI with *K. pneumoniae* resulted in higher expression levels. These limited interactions between TBI and *K. pneumoniae* warrant further exploration in future studies. For example, CCL2 is one of the key chemokines induced by severe brain injury in both experimental models and TBI patients [74]. It is also remarkably upregulated in response to K. pneumoniae infection, at least in lung tissue [75], where it plays a distinct role in the recruitment of CCR2 + monocytes necessary for bacterial clearance [76]. Whether a bacterial infection superimposed upon a injured brain alters CCL2/CCR2-mediated monocyte activity remains unclear.

These TBI-induced gene expression changes were likely associated with the robust cellular immune response detected by IF staining at 7 d post-infection, including infiltration of MPO + cells (presumably neutrophils) and reactivity of GFAP (presumably astrocytes) in the injured cortex and hippocampus. The extent of this staining was similar in both *K. pneumoniae* and vehicle-treated TBI groups, suggesting that the detected effects of *K. pneumoniae* on *Tnfα, Ccl2, Hmox1* and *Arg1* gene expression were insufficient to drive a differential cellular immune response. Similarly, while pronounced tissue loss was evident in the ipsilateral cortex and hippocampus of TBI mice, there was no additive effect of *K. pneumoniae* on neuropathology. Together, these findings are contrary to our initial hypothesis, and indicate that the pathophysiological responses to a sequential brain injury and lung infection are not necessarily interactive.

Finally, we investigated the fecal microbiome across the time course—from pre-injury up to 7 days post-infection, considering increasing evidence of complex brain-gut connections as well as aberrant microbiome profiles as a consequence of TBI [38, 39]. *K. pneumoniae*-induced pneumonia, as well as several other respiratory diseases, have also been reported to alter the gut microbiome, for example, by reducing the relative abundance of *Bifidobacterium* and *Clostridium* [40]. However, somewhat surprisingly, we did not detect a robust effect of either TBI or *K. pneumoniae* on fecal bacterial diversity or composition. This lack of an effect perhaps suggests that the moderate dose of *K. pneumoniae*, and transient nature of the subsequent lung infection, may have been insufficient to induce significant consequences for the gut. It is also worth noting that mice were group-housed as same-sex littermates, with each cage containing a mix of experimental groups to control for potential litter effects. However, as mice are coprophagous, cross-contamination of fecal material between mice from different experimental groups may have masked possible injury or infection effects.

Altogether, this study highlights some of the challenges associated with modeling complex conditions such as concurrent bacterial infections in brain-injured individuals. It is particularly difficult to achieve an optimal balance between a robust, reproducible infection which induces sufficient pathology in an experimental setting, while taking into consideration ethical research principles of refinement and reduction [77]. The rapid clearance of *K. pneumoniae* bacteria by immunocompetent mice limits the time window for the assessment of potential interactions between infection and neurotrauma, while also impacting the potential clinical relevance of the model. A future possibility would be to employ the model in neutropenic or immunocompromised mice [e.g., 44], to more closely mimic the altered immune status of a severely-injured patient in the ICU. Alternatively, a higher bacterial load could be trialed incorporating the use of antibiotics or other clinically-relevant treatment paradigms [e.g., 64], to better model a patient’s complex medical management.

Our experimental groups contained both male and female mice, and we typically failed to detect any sexually dimorphic effects; although this may be influenced by a lack of statistical power for some experiments when the groups were sub-divided by sex. Sex differences in acute neuroinflammation after experimental TBI have been reported; for example, Doran and colleagues found an increase in peripheral myeloid cells infiltrating the brain in males, alongside increased resident microglial proliferation [78]. Sex differences exist in terms of baseline respiratory physiology, and males typically fairing worse than females in models of respiratory disease [79]. The survival of mice after *K. pneumoniae* infection is reportedly sex-dependent, mediated by gonadal hormones and/or oxidative stress responses [67, 80, 81]. In our model, we anecdotally observed that males exhibited exacerbated sickness symptoms (e.g., scruffy fur, hunched posture, immobility) within hours after *K. pneumoniae* inoculation. However, this did not manifest as statistically significant differences between the sexes in terms of percentage body weight loss or reduced locomotor activity.

Finally, further investigation of a more chronic time course may reveal novel insights into the potential long-term consequences of TBI and infection, even if the infection is transient and contained. For example, patients who survive acute infections may be vulnerable to long-term complications including neurocognitive dysfunction [82, 83]. In a mouse model of intranasal *K. pneumoniae*-induced pneumosepsis treated with ceftriaxone, even after the resolution of infection, surviving mice showed deficits in exploratory locomotor behavior associated with persistent brain inflammatory gene expression [64]. Thus, ongoing work in our laboratory is now exploring the potential long-term consequences of combined TBI and *K. pneumoniae* infections on neuropathology and neurobehavioral outcomes.

## Conclusion

Overall, this study found that a moderate-severe TBI combined with a moderate inoculation of *K. pneumoniae* into the lungs had largely independent effects on brain and lung pathophysiology, respectively. The consequences of *K. pneumoniae* infection were predominantly restricted to a robust, acute, and transient pro-inflammatory response in the lungs, alongside a lower magnitude increase in serum cytokines. *K. pneumoniae* appeared to influence the expression of a subset of immune-related genes in the injured cortex and hippocampus, but these changes did not translate into any effects on the extent of neuropathology. While experimental paradigms of combined TBI and live infection models are rare, studies incorporating post-TBI infection with *Citrobacter rodentium*, *Pseudomonas aeruginosa*, or *Streptococcus pneumoniae* have all reported that the dual insult worsens neuroinflammation and mortality [32, 33, 38]. Of note, these studies all incorporated a moderate or severe TBI model, whereas other studies have suggested that mild TBI may conversely be protective against *P. aeruginosa* pneumonia [71, 72]. A more complete understanding of combined neurotrauma and infection insults is necessary to ensure that model systems can generate data of clinical relevance [31]. Despite our findings in this study, most clinical evidence indicates that pulmonary infections after severe TBI represent a key determinant of prognosis, and both the prevention and treatment of infections are high priorities in hospital settings [17, 63, 84]. Further, the growing global prevalence of antimicrobial resistance and hypervirulence in *Enterobacteriaceae* such as *Kp* is a serious public health concern, associated with poorer outcomes, higher mortality, and limited treatment options [85–87]. Further research in this space is therefore warranted.

### Electronic supplementary material

Below is the link to the electronic supplementary material.

Supplementary Material 1

## Data Availability

The datasets supporting the conclusions of this article are available in the Monash Bridges repository (www.bridges.monash.edu), doi: 10.26180/25058048. (Note: accessible upon publication).

## References

[1] Maas AIR, Menon DK, Manley GT (2022). Traumatic brain injury: progress and challenges in prevention, clinical care, and research. Lancet Neurol.

[2] van Dijck J, Mostert CQB, Greeven APA (2020). Functional outcome, in-hospital healthcare consumption and in-hospital costs for hospitalised traumatic brain injury patients: a Dutch prospective multicentre study. Acta Neurochir (Wien).

[3] Van Deynse H, Van Belleghem G, Lauwaert D (2019). The incremental cost of traumatic brain injury during the first year after a road traffic accident. Brain Inj.

[4] Kumar RG, Kesinger MR, Juengst SB (2020). Effects of hospital-acquired pneumonia on long-term recovery and hospital resource utilization following moderate to severe traumatic brain injury. J Trauma Acute Care Surg.

[5] Rincón-Ferrari MD, Flores-Cordero JM, Leal-Noval SR (2004). Impact of ventilator-associated pneumonia in patients with severe head injury. J Trauma.

[6] Kesinger MR, Kumar RG, Wagner AK (2015). Hospital-acquired pneumonia is an independent predictor of poor global outcome in severe traumatic brain injury up to 5 years after discharge. J Trauma Acute Care Surg.

[7] Yang CC, Shih NC, Chang WC (2011). Long-term medical utilization following ventilator-associated pneumonia in acute stroke and traumatic brain injury patients: a case-control study. BMC Health Serv Res.

[8] Schirmer-Mikalsen K, Moen KG, Skandsen T (2013). Intensive care and traumatic brain injury after the introduction of a treatment protocol: a prospective study. Acta Anaesthesiol Scand.

[9] Piek J, Chesnut RM, Marshall LF (1992). Extracranial complications of severe head injury. J Neurosurg.

[10] Corral L, Javierre CF, Ventura JL (2012). Impact of non-neurological complications in severe traumatic brain injury outcome. Crit Care.

[11] Woratyla SP, Morgan AS, Mackay L (1995). Factors associated with early onset pneumonia in the severely brain-injured patient. Conn Med.

[12] Hsieh AH, Bishop MJ, Kubilis PS (1992). Pneumonia following closed head injury. Am Rev Respir Dis.

[13] Chen S, Gao G, Xia Y (2023). Incidence rate and risk factors of ventilator-associated pneumonia in patients with traumatic brain injury: a systematic review and meta-analysis of observational studies. J Thorac Disease.

[14] Li Y, Liu C, Xiao W (2020). Incidence, risk factors, and outcomes of Ventilator-Associated Pneumonia in Traumatic Brain Injury: a Meta-analysis. Neurocrit Care.

[15] Griffin GD (2011). The injured brain: TBI, mTBI, the immune system, and infection: connecting the dots. Mil Med.

[16] Poole D, Chieregato A, Langer M (2014). Systematic review of the literature and evidence-based recommendations for antibiotic prophylaxis in trauma: results from an Italian consensus of experts. PLoS ONE.

[17] Kourbeti IS, Vakis AF, Papadakis JA et al. Infections in traumatic brain injury patients. Clinical microbiology and infection: the official publication of the European Society of Clinical Microbiology and Infectious diseases. 2012;18(4):359–64. 10.1111/j.1469-0691.2011.03625.x 21851488

[18] Tunthanathip T, Tepaamondej N (2019). Factors associated with surgical site infection in blast-induced traumatic brain injury. Chin Med J.

[19] Gahagen RE, Beardsley AL, Maue DK et al. Early-Onset Ventilator-Associated Pneumonia in Pediatric Severe Traumatic Brain Injury. Neurocritical care. 2023. 10.1007/s12028-022-01663-4 36635493

[20] Al Qasem MA, Algarni AM, Al Bshabshe A (2023). The microbiological profile of isolates recovered from ICU patients with traumatic brain injuries at a tertiary care center, Southern Region, Saudi Arabia. J Infect Public Health.

[21] Hamele M, Stockmann C, Cirulis M (2016). Ventilator-Associated Pneumonia in Pediatric Traumatic Brain Injury. J Neurotrauma.

[22] McDaniel DK, Allen IC (2019). Using Klebsiella pneumoniae to Model Acute Lung inflammation in mice. Methods Mol Biol.

[23] Zembower NR, Zhu A, Malczynski M (2017). Klebsiella pneumoniae carbapenemase-producing K. pneumoniae (KPC-KP) in brain and spinal cord injury patients: potential for prolonged colonization. Spinal Cord.

[24] Peleg AY, Hooper DC (2010). Hospital-acquired infections due to gram-negative bacteria. N Engl J Med.

[25] Keynan Y, Rubinstein E (2007). The changing face of Klebsiella pneumoniae infections in the community. Int J Antimicrob Agents.

[26] Santajit S, Indrawattana N (2016). Mechanisms of Antimicrobial Resistance in ESKAPE pathogens. Biomed Res Int.

[27] Tacconelli E, Carrara E, Savoldi A (2018). Discovery, research, and development of new antibiotics: the WHO priority list of antibiotic-resistant bacteria and tuberculosis. Lancet Infect Dis.

[28] Ritzel RM, Doran SJ, Barrett JP (2018). Chronic alterations in systemic Immune function after traumatic brain Injury. J Neurotrauma.

[29] Farag E, Machado S, Argalious M (2023). Multiorgan talks in the presence of brain injury. Curr Opin Anaesthesiol.

[30] Matin N, Sarhadi K, Crooks CP (2022). Brain-lung crosstalk: management of concomitant severe Acute Brain Injury and Acute Respiratory Distress Syndrome. Curr Treat Options Neurol.

[31] Gandasasmita N, Li J, Loane DJ et al. Experimental models of Hospital-Acquired infections after traumatic Brain Injury: challenges and opportunities. J Neurotrauma. 2023. 10.1089/neu.2023.0453 37885226

[32] Pittet JF, Hu PJ, Honavar J (2021). Estrogen alleviates sex-dependent differences in Lung Bacterial Clearance and mortality secondary to bacterial pneumonia after traumatic brain Injury. J Neurotrauma.

[33] Doran SJ, Henry RJ, Shirey KA et al. Early or late bacterial lung infection increases Mortality after Traumatic Brain Injury in male mice and chronically impairs Monocyte Innate Immune function. Crit Care Med. 2020. 10.1097/CCM.0000000000004273 PMC7541908 32149839

[34] Vermeij JD, Aslami H, Fluiter K (2013). Traumatic brain injury in rats induces lung injury and systemic immune suppression. J Neurotrauma.

[35] Lin YW, Zhou QT, Cheah SE et al. Pharmacokinetics/Pharmacodynamics of Pulmonary Delivery of Colistin against Pseudomonas aeruginosa in a mouse lung infection model. Antimicrob Agents Chemother. 2017;61(3). 10.1128/AAC.02025-16 PMC5328518 28031207

[36] Hackstein H, Kranz S, Lippitsch A, et al. Modulation of respiratory dendritic cells during Klesiella pneumonia infection. Respir Res. 2013;14(91). 10.1186/465-9921-14-91. 10.1186/1465-9921-14-91 PMC3848864 24044871

[37] Dietert K, Gutbier B, Wienhold SM (2017). Spectrum of pathogen- and model-specific histopathologies in mouse models of acute pneumonia. PLoS ONE.

[38] Ma EL, Smith AD, Desai N (2017). Bidirectional brain-gut interactions and chronic pathological changes after traumatic brain injury in mice. Brain Behav Immun.

[39] Hanscom M, Loane DJ, Shea-Donohue T. Brain-gut axis dysfunction in the pathogenesis of traumatic brain injury. J Clin Investig. 2021;131(12). 10.1172/JCI143777 PMC8203445 34128471

[40] Jiang Q, Xu Q, Kenéz Á (2022). Klebsiella pneumoniae infection is associated with alterations in the gut microbiome and lung metabolome. Microbiol Res.

[41] Sharma R, Casillas-Espinosa PM, Dill LK (2022). Pediatric traumatic brain injury and a subsequent transient immune challenge independently influenced chronic outcomes in male mice. Brain Behav Immun.

[42] Hussein M, Jasim R, Gocol H (2023). Comparative proteomics of outer membrane vesicles from Polymyxin-Susceptible and extremely drug-resistant Klebsiella pneumoniae. mSphere.

[43] Lin YW, Zhou Q, Onufrak NJ et al. Aerosolized polymyxin B for treatment of respiratory tract infections: determination of pharmacokinetic-pharmacodynamic indices for Aerosolized Polymyxin B against Pseudomonas aeruginosa in a mouse lung infection model. Antimicrob Agents Chemother. 2017;61(8). 10.1128/AAC.00211-17 PMC5527630 28559256

[44] Landersdorfer CB, Wang J, Wirth V (2018). Pharmacokinetics/pharmacodynamics of systemically administered polymyxin B against Klebsiella pneumoniae in mouse thigh and lung infection models. J Antimicrob Chemother.

[45] Raftery AL, O’Brien CA, Harris NL (2023). Development of severe colitis is associated with lung inflammation and pathology. Front Immunol.

[46] Kluver H, Barrera E (1953). A method for the combined staining of cells and fibers in the nervous system. J Neuropathol Exp Neurol.

[47] Gundersen HJG, Jensen EB (1987). The efficiency of systematic sampling in stereology and its prediction. J Microsc.

[48] Sharma R, Zamani A, Dill LK (2021). A systemic immune challenge to model hospital-acquired infections independently regulates immune responses after pediatric traumatic brain injury. J Neuroinflammation.

[49] Wickramasinghe LC, Lau M, Deliyanti D (2020). Lung and Eye Disease develop concurrently in Supplemental Oxygen-exposed neonatal mice. Am J Pathol.

[50] Rapin A, Pattaroni C, Marsland BJ (2017). Microbiota Analysis using an Illumina MiSeq platform to sequence 16S rRNA genes. Curr Protocols Mouse Biology.

[51] Pattaroni C, Macowan M, Chatzis R (2022). Early life inter-kingdom interactions shape the immunological environment of the airways. Microbiome.

[52] Callahan BJ, McMurdie PJ, Rosen MJ (2016). DADA2: high-resolution sample inference from Illumina amplicon data. Nat Methods.

[53] Eren AM, Vineis JH, Morrison HG (2013). A filtering method to generate high quality short reads using illumina paired-end technology. PLoS ONE.

[54] Martin M. Cutadapt removes adapter sequences from high-throughput sequencing reads. 2011. 2011;17(1):3.

[55] Callahan BJ, Sankaran K, Fukuyama JA (2016). Bioconductor Workflow for Microbiome Data Analysis: from raw reads to community analyses. F1000Research.

[56] Davis NM, Proctor DM, Holmes SP (2018). Simple statistical identification and removal of contaminant sequences in marker-gene and metagenomics data. Microbiome.

[57] Li D (2018). hillR: taxonomic, functional, and phylogenetic diversity and similarity through Hill Numbers. J Open Source Softw.

[58] Paulson JN, Stine OC, Bravo HC (2013). Differential abundance analysis for microbial marker-gene surveys. Nat Methods.

[59] McMurdie PJ, Holmes S (2013). Phyloseq: an R package for reproducible interactive analysis and graphics of microbiome census data. PLoS ONE.

[60] Noguchi K, Gel YR, Brunner E (2012). nparLD: an R Software Package for the Nonparametric Analysis of Longitudinal Data in Factorial experiments. J Stat Softw.

[61] Ritchie ME, Phipson B, Wu D (2015). Limma powers differential expression analyses for RNA-sequencing and microarray studies. Nucleic Acids Res.

[62] Kourbeti IS, Vakis AF, Ziakas P (2015). Infections in patients undergoing craniotomy: risk factors associated with post-craniotomy meningitis. J Neurosurg.

[63] Zhang X, Zhou H, Shen H (2022). Pulmonary infection in traumatic brain injury patients undergoing tracheostomy: predicators and nursing care. BMC Pulm Med.

[64] Denstaedt SJ, Spencer-Segal JL, Newstead M (2020). Persistent neuroinflammation and brain-specific Immune Priming in a Novel Survival Model of Murine Pneumosepsis. Shock.

[65] Elemary NM, Emara MM, Elhady Tahoun AA (2023). Immune Response and Pathophysiological features of Klebsiella Pneumoniae in mice. JPMA J Pakistan Med Association.

[66] Dill LK, Teymornejad S, Sharma R et al. Modulating chronic outcomes after pediatric traumatic brain injury: distinct effects of social and environmental enrichment. Exp Neurol. 2023:114407. 10.1016/j.expneurol.2023.114407 37059414

[67] Mikerov AN, Cooper TK, Wang G (2011). Histopathologic evaluation of lung and extrapulmonary tissues show sex differences in Klebsiella pneumoniae - infected mice under different exposure conditions. Int J Physiol Pathophysiology Pharmacol.

[68] van der Geest R, Fan H, Peñaloza HF (2023). Phagocytosis is a primary determinant of pulmonary clearance of clinical Klebsiella pneumoniae isolates. Front Cell Infect Microbiol.

[69] Rammaert B, Goyet S, Beauté J (2012). Klebsiella pneumoniae related community-acquired acute lower respiratory infections in Cambodia: clinical characteristics and treatment. BMC Infect Dis.

[70] Sharma R, Shultz SR, Robinson MJ et al. Infections after a traumatic brain injury: the complex interplay between the immune and neurological systems. Brain, Behavior, and Immunity. 2019;79:63–74. 10.1016/j.bbi.2019.04.034 31029794

[71] Vaickus M, Hsieh T, Kintsurashvili E (2019). Mild traumatic brain Injury in mice beneficially alters lung NK1R and structural protein expression to Enhance Survival after Pseudomonas aeruginosa infection. Am J Pathol.

[72] Ruan F, Chen J, Yang J, MILD TRAUMATIC BRAIN INJURY ATTENUATES PNEUMONIA-INDUCED LUNG INJURY BY MODULATIONS OF ALVEOLAR MACROPHAGE BACTERICIDAL ACTIVITY AND M1 POLARIZATION (2022). Shock.

[73] Yang S, Stepien D, Hanseman D (2014). Substance P mediates reduced pneumonia rates after traumatic brain injury. Crit Care Med.

[74] Semple BD, Bye N, Rancan M (2010). Role of CCL2 (MCP-1) in traumatic brain injury (TBI): evidence from severe TBI patients and CCL2-/- mice. JCBFM.

[75] Lei L, Zhang X, Yang R (2022). Host Immune Response to Clinical Hypervirulent Klebsiella pneumoniae pulmonary infections via Transcriptome Analysis. J Immunol Res.

[76] Xiong H, Carter RA, Leiner IM (2015). Distinct contributions of neutrophils and CCR2 + monocytes to Pulmonary Clearance of different Klebsiella pneumoniae strains. Infect Immun.

[77] Kiani AK, Pheby D, Henehan G (2022). Ethical considerations regarding animal experimentation. J Prev Med Hyg.

[78] Doran SJ, Ritzel RM, Glaser EP (2019). Sex differences in Acute Neuroinflammation after Experimental Traumatic Brain Injury are mediated by infiltrating myeloid cells. J Neurotrauma.

[79] Card JW, Zeldin DC (2009). Hormonal influences on lung function and response to environmental agents: lessons from animal models of respiratory disease. Proc Am Thorac Soc.

[80] Durrani F, Phelps DS, Weisz J (2012). Gonadal hormones and oxidative stress interaction differentially affects survival of male and female mice after lung Klebsiella pneumoniae infection. Exp Lung Res.

[81] Mikerov AN, Gan X, Umstead TM (2008). Sex differences in the impact of ozone on survival and alveolar macrophage function of mice after Klebsiella pneumoniae infection. Respir Res.

[82] Andonegui G, Zelinski EL, Schubert CL et al. Targeting inflammatory monocytes in sepsis-associated encephalopathy and long-term cognitive impairment. JCI Insight. 2018;3(9). 10.1172/jci.insight.99364 PMC6012517 29720578

[83] Hernandez-Ruiz V, Letenneur L, Fülöp T (2022). Infectious diseases and cognition: do we have to worry?. Neurol Sciences: Official J Italian Neurol Soc Italian Soc Clin Neurophysiol.

[84] Russo E, Antonini MV, Sica A et al. Infection-related Ventilator-Associated complications in critically ill patients with trauma: a retrospective analysis. Antibiot (Basel Switzerland). 2023;12(1). 10.3390/antibiotics12010176 PMC9854794 36671377

[85] Gugliandolo A, Caio C, Mezzatesta ML (2017). Successful ceftazidime-avibactam treatment of MDR-KPC-positive Klebsiella pneumoniae infection in a patient with traumatic brain injury: a case report. Medicine.

[86] Silvester R, Madhavan A, Kokkat A (2022). Global surveillance of antimicrobial resistance and hypervirulence in Klebsiella pneumoniae from LMICs: an in-silico approach. Sci Total Environ.

[87] Salawudeen A, Raji YE, Jibo GG (2023). Epidemiology of multidrug-resistant Klebsiella pneumoniae infection in clinical setting in South-Eastern Asia: a systematic review and meta-analysis. Antimicrob Resist Infect Control.

