# Supplementary figures and images for: Modelling lung infection with Klebsiella pneumoniae after murine traumatic brain injury
Source: J Neuroinflammation. 2024 May 8;21:122. doi: 10.1186/s12974-024-03093-9 (PMC11080247; doi:10.1186/s12974-024-03093-9)

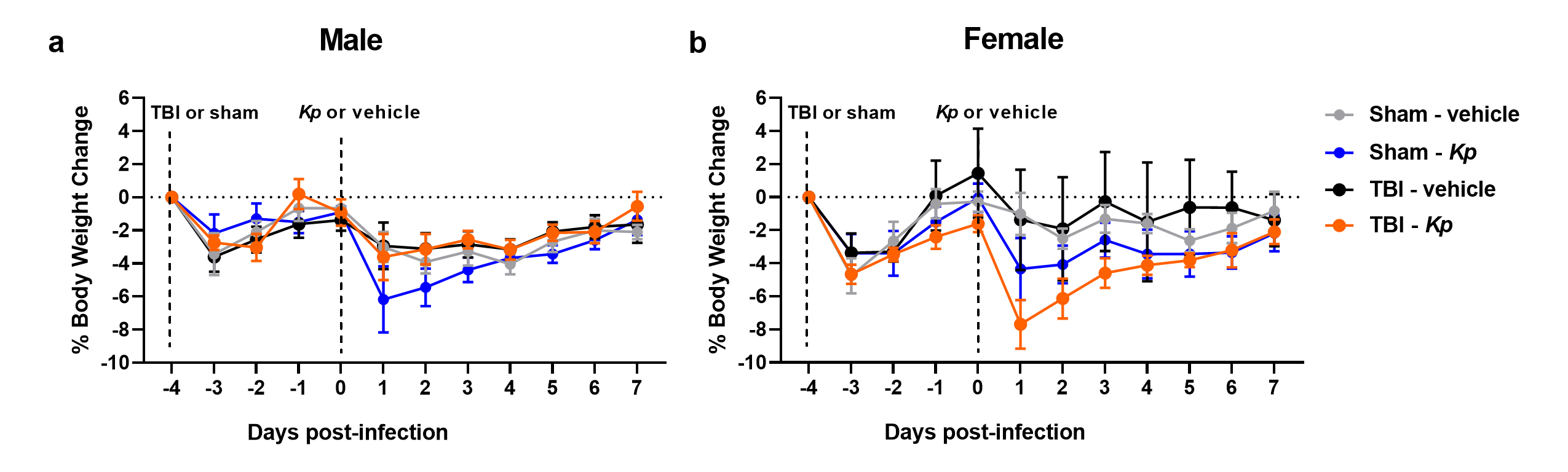

Supplement: Supplementary file 1 — Supplementary Material 1 [file 12974_2024_3093_MOESM1_ESM.tif]
